# Transcriptional dynamics in the protozoan parasite *Sarcocystis neurona* and mammalian host cells after treatment with a specific inhibitor of apicomplexan mRNA polyadenylation

**DOI:** 10.1371/journal.pone.0259109

**Published:** 2021-10-28

**Authors:** Arthur G. Hunt, Daniel K. Howe, Ashley Brown, Michelle Yeargan

**Affiliations:** 1 Department of Plant and Soil Sciences, University of Kentucky, Lexington, KY, United States of America; 2 Department of Veterinary Science, University of Kentucky, Lexington, KY, United States of America; University of Melbourne, AUSTRALIA

## Abstract

In recent years, a class of chemical compounds (benzoxaboroles) that are active against a range of parasites has been shown to target mRNA polyadenylation by inhibiting the activity of CPSF73, the endonucleolytic core of the eukaryotic polyadenylation complex. One particular compound, termed AN3661, is active against several apicomplexan parasites that cause disease in humans. In this study, we report that AN3661 is active against an apicomplexan that causes disease in horses and marine mammals (*Sarcocystis neurona*), with an approximate IC_50_ value of 14.99 nM. Consistent with the reported mode of action of AN3661 against other apicomplexans, *S*. *neurona* mutants resistant to AN3661 had an alteration in CPSF73 that was identical to a mutation previously documented in AN3661-resistant *Toxoplasma gondii* and *Plasmodium falciparum*. AN3661 had a wide-ranging effect on poly(A) site choice in *S*. *neurona*, with more than half of all expressed genes showing some alteration in mRNA 3’ ends. This was accompanied by changes in the relative expression of more than 25% of *S*. *neurona* genes and an overall 5-fold reduction of *S*. *neurona* transcripts in infected cells. In contrast, AN3661 had no discernible effect on poly(A) site choice or gene expression in the host cells. These transcriptomic studies indicate that AN3661 is exceedingly specific for the parasite CPSF73 protein, and has the potential to augment other therapies for the control of apicomplexan parasites in domestic animals.

## Introduction

The phylum Apicomplexa encompasses a very broad group of obligate intracellular parasites that are a significant cause of disease worldwide [1]. Several of these are important human pathogens, such as *Plasmodium* spp. and *Toxoplasma gondii*. As well, multiple members of this phylum cause disease in domestic animals and have a significant economic impact on agriculture. Both *Theileria* spp. and *Babesia* spp. are tick-borne haemoprotozoan pathogens that infect and cause disease in cattle and horses. Species of *Eimeria* are enteric parasites that cause diarrheal diseases in a variety of animals and are especially detrimental to the poultry industry. *Neospora caninum* was initially described as a neurologic pathogen of canids, but has subsequently been identified as a major cause of reproductive failure in cattle. *Sarcocystis neurona* is an important cause of the neurologic disease equine protozoal myeloencephalitis (EPM) and an emerging pathogen of marine mammals. While the economic impact of these pathogens is difficult to assess, the annual global cost of neosporosis alone has been estimated at greater than $1 billion dollars [2].

Although vaccination is an option for reducing coccidiosis caused by *Eimeria* spp. in poultry, vaccines against other coccidian parasites have generally proven ineffective. Consequently, chemotherapy has been the standard approach to try to control infection and disease caused by these parasites (reviewed in [3–5]). Drug combinations that target folate metabolism, such as pyrimethamine and a sulfonamide, have been used for decades to treat infections by multiple apicomplexan parasites, including the coccidia. However, the routine use of anti-folate drugs to treat coccidial infections has been hindered by several factors, including the duration of treatment (6 months for horses with EPM) and associated toxicities (anemia, leukopenia, teratogenicity). The benzeneacetonitrile compounds diclazuril, ponazuril, and toltrazuril are effective treatments for agricultural animals infected with coccidian parasites. However, concerns about drug residues in food products and the ease in developing resistance have limited the use of the benzeneacetonitrile drugs in agricultural animals [3–5]. Anticoccidial medications are used extensively against *Eimeria* and are quite commonly incorporated into poultry feeds. A variety of drugs have been employed for this purpose, but ionophore antibiotics, such as monensin, are currently the most widely used anticoccidials to control coccidiosis (reviewed in [6]). While resistance to the ionophore drugs has been slower to occur, reports now indicate that monensin-resistant isolates of *Eimeria* are widespread [6]. Consequently, the continued use of existing anticoccidial drugs to control these parasites might not be a viable long-term option. Taken together, these considerations reveal an ongoing need for affective alternative therapies for coccidian diseases in agricultural animals.

Recently, it was reported that a member of a class of benzoxaboroles, termed AN3661, inhibits growth of *Plasmodium falciparum* [7], *T*. *gondii* [8], and *Cryptosporidium parvum* [9]. The demonstrated mode of action of AN3661 is novel, in that it targets the 73 kDa subunit of the Cleavage and Polyadenylation Specificity Factor, or CPSF73, of the apicomplexan polyadenylation complex. In this report, we show that AN3661 similarly inhibited the coccidian parasite *S*. *neurona*. We also show that AN3661 had a decided effect on transcriptional dynamics of *S*. *neurona*, while drug treatment of the mammalian host cells had little impact on gene expression and mRNA polyadenylation in these cells. Together, these results expand the list of apicomplexans species that are susceptible to AN3661, and they reveal that the drug is an exceedingly selective inhibitor, with little discernible impact on the gene expression dynamics of the mammalian host.

## Materials and methods

### Parasite cultures, DNA isolation, and parasite growth assays

*S*. *neurona* strains (wild-type strain SN3.E1, YFP-expressing clone F9F, and AN3661-resistant clones were propagated by serial passage in monolayers of BT cells, as described [10]. Upon lysis of the infected monolayers, extracellular merozoites were harvested by passing through 23 and 25-gauge needles and filter-purified to remove host cell debris. Merozoites were pelleted and stored at -80°C until used for DNA or RNA isolation.

For parasite growth assays, YFP-expressing *S*. *neurona* was used according to procedures described previously [11]. Freshly-released merozoites purified from host cell debris were resuspended in culture medium without phenol red, and 96-well plates containing BT monolayers were inoculated with 4 x 10^4^ parasites per well, eight replicates (wells) per treatment. Control wells containing no parasites or no drug were included to account for background fluorescence and relative growth, respectively. Non-invaded merozoites were washed out after 2 hrs, and medium containing the appropriate dilution of AN3661 was added back to the wells and left for the duration of the growth assay. The plates were incubated at 37°C for 4 days, and fluorescence was measured using a Synergy H1 plate reader (BioTek, Winooski, VT, USA). The relative fluorescence units (RFUs) in the no-parasite wells was subtracted from the no-drug control and treatment wells, and growth of *S*. *neurona* in the presence of AN3661 was determined by comparing the RFUs in treated wells with those from non-treated control wells. The half-maximal inhibitory concentration (IC_50_) was determined by regression analysis (GraphPad Prism v. 9).

### Isolation and sequence analysis of AN3661-resistant clones of *S*. *neurona*

*Sarcocystis neurona* strain SN3.E1 was mutagenized with 2 mM N-ethyl-N-nitrosourea (ENU), as described previously for *T*. *gondii* [12]. The mutagenized culture was allowed to recover and expand for 3 days before addition of 90 nM AN3661 to select for mutant parasites that had become resistant to the drug. The *S*. *neurona* culture was maintained in medium containing AN3661 until the parasites disrupted the host cell monolayer (approximately 5 weeks), and single-cell clones of AN3661-resistant parasites were isolated in 96-well plates and expanded in the presence of drug for further analyses, as described [13].

The full-length coding region of SnCPSF73 was amplified in sections and sequenced using the primers listed in S1 Table. Sequences obtained from the AN3661-resistant clones were aligned with the SnCPSF73 gene from the SN3.E1 reference genome (SN3_01500330) to identify nucleotide polymorphisms. The SnCPSF73 amino acid sequence was further aligned to the human (NP_057291.1), *Arabidopsis* (At1g61010.1), *T*. *gondii* (TGME49_285200-t26_1), and *P*. *falciparum* (PF3D7_1438500.1) CPSF73 amino acid sequences so as to display commonalities in mutations that arise in AN3661-resistant *S*. *neurona* clones.

### Poly(A) site profiling

*S*. *neurona* strain SN3.E1 was inoculated onto BT host cell monolayers grown in 6-well plates. After 48 hrs of parasite development, triplicate wells (each well representing a biological replicate) were incubated in fresh media with or without 90 nM AN3661 for an additional 24 hrs. The infected BT monolayers were then harvested from the wells using cell scrapers and centrifuged at 1100 x g for 10 min at 4°C. The cell pellets were washed 1x with ice-cold PBS and then stored at -80°C until used for RNA isolation. To conduct poly(A) site profiling in the non-infected host cells, confluent monolayers of BT cells grown in 6-well plates, triplicate wells were similarly treated with or without AN3661 for 24 hrs, and the cells harvested for RNA isolation.

Total RNA was isolated by adding 1mL Trizol to cell pellets, incubating for 5 minutes at room temperature, then adding 200 μL chloroform and incubating for an additional 3 minutes at room temperature. Samples were centrifuged at 13,200 x g for 15 minutes, and the aqueous layer was transferred to a fresh tube. 500 μL of isopropanol was added to the sample and incubated overnight at -20°C. The tubes were centrifuged at 13,200 x g for 10 minutes, and the RNA pellet was washed with 1 mL 75% EtOH. The pellet was air dried for 5–10 minutes and resuspended in RNase free water.

Libraries for genome-wide poly(A) site profiling was performed following the procedures described in Ma *et al*. [14] and Pati *et al*. [15]. So-called poly(A) tag (PAT-Seq) libraries were sequenced on an Illumina HiSeq instrument; the sequencing data are available under Bioproject PRJNA713353. Sequencing reads were trimmed, demultiplexed, and mapped to the respective genomes using CLC Genomics Workbench (latest version used was 20.0.4); the results of these analyses are summarized in S1 File. Subsequent analyses were performed following the pipelines described in Bell *et al*. [16] and de Lorenzo *et al*. [17]. This pipeline is described in detail in S2 File.

Gene expression was estimated by mapping individual PAT-Seq reads to the *S*. *neurona* or *B*. *taurus* genome annotations using the RNASeq tool in CLC Genomics Workbench. The process is described in detail in S2 File.

### Host cell cytotoxicity assay

The cytotoxicity of AN3661 for the bovine host cells was determined by release of lactate dehydrogenase (LDH) from treated cells using a colorimetric assay (Pierce LDH Cytotoxicity Assay, Thermo Scientific). Briefly, BT cells seeded in a 96-well plate were treated in triplicate in increasing concentrations of AN3661. The plate was incubated for 24 hours, and LDH release from the treated cells was determined spectrophotometrically by subtracting the 680 nm absorbance (background) from the 490 nm absorbance following the protocol provided by the manufacturer. The maximum release of LDH was determined by addition of lysis buffer (provided by the manufacturer) to non-treated triplicate wells of BT cells. The LDH positive control was provided by the manufacturer.

## Results

### AN3661 inhibits the growth of *Sarcocystis neurona* and *Neospora caninum*

**Fig 1:**
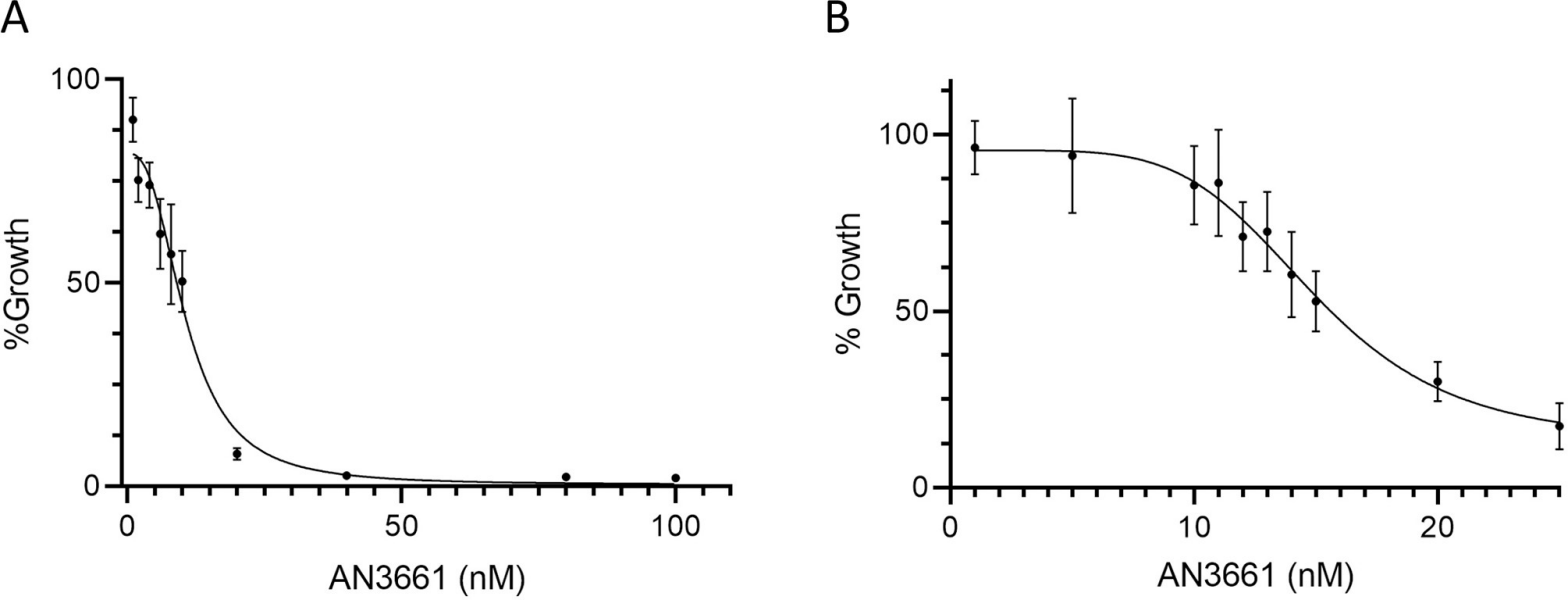
Growth inhibition of *S*. *neurona* by AN3661. An *S*. *neurona* clone expressing YFP was seeded into 96-well plates containing monolayers of BT host cells and incubated for 4 days (*S*. *neurona*) in the presence of increasing concentrations of AN3661 (eight replicates/concentration). Parasite growth inhibition was based on relative fluorescence of treated wells compared to control wells containing no drug, and the IC_50_ was determined by regression analysis. (A) Parasite growth in AN3661 concentrations between 1 nM and 100 nM suggested an IC_50_ of 10.68 nM, with parasite growth virtually halted at concentrations greater than 50 nM. (B) Parasite growth across a narrow range of AN3661 concentrations indicated an IC_50_ of 14.99 nM.

To test the hypothesis that AN3661 inhibits *S*. *neurona*, growth assays were performed using a parasite clone expressing YFP, as described previously [11,18]. Our preliminary growth assays suggested that AN3661 inhibited *S*. *neurona* at low nanomolar concentrations (data not shown). Based on these initial assays, *S*. *neurona* growth was examined in the presence of AN3661 over a range of 1 nM to 100 nM (Fig 1A). This showed that *S*. *neurona* is very sensitive to AN3661, with an estimated IC_50_ of 10.68 nM and nearly all parasite growth inhibited at concentrations greater than 50 nM (Fig 1A). To obtain a better estimate of the AN3661 IC_50_ for *S*. *neurona*, the growth assay was repeated using a narrower range of drug concentration (1–25 nM). This assay revealed an IC_50_ of 14.99 nm (Fig 1B), a concentration comparable to the IC_50_ described for two isolates of *P*. *falciparum* [7], but significantly less than the IC_50_ reported for *C*. *parvum* (80 nM [9]) and *T*. *gondii* (900 nM [8]).

### AN3661-resistant *S*. *neurona* have mutations near the active site of CPSF73

**Fig 2:**
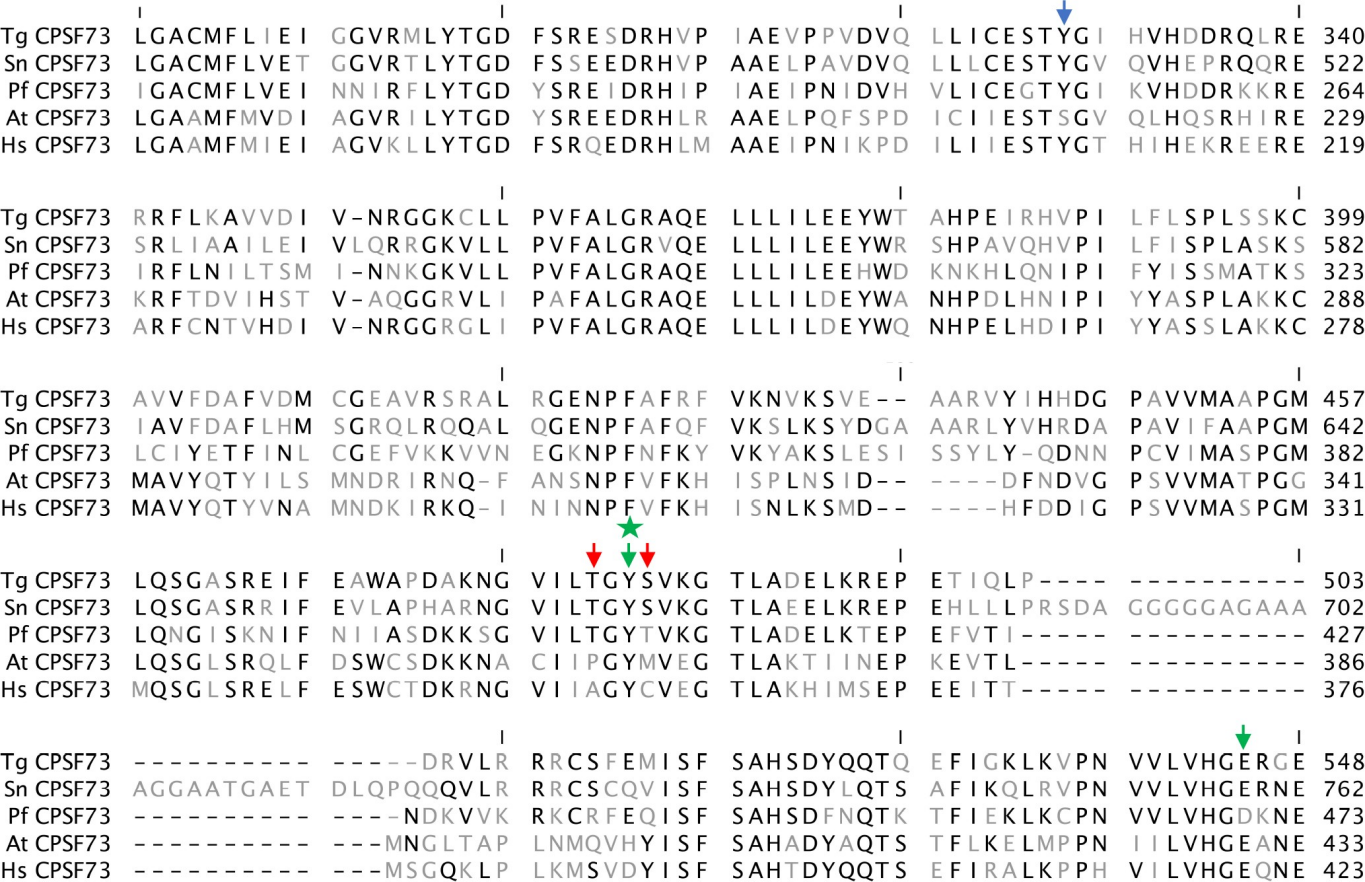
Amino acid sequence alignments of orthologs of CPSF73. Alignments were performed using the default settings in CLC Genomics Workbench. Amino acid residues that are identical in all five sequences are shown in black, and other residues in gray. Residues that are altered in *T*. *gondii* AN3661-resistant mutants are denoted with a blue arrow, those altered in resistant *P*. *falciparum* mutants in red, and those altered in both *T*. *gondii* and *P*. *falciparum* mutants in green. The green star denotes the position (Y668 in the *S*. *neurona* protein, corresponding to Y483 of the *T*. *gondii* CPSF73) that is altered in the five AN3661-resistance *S*. *neurona* clones. The alignment is truncated, focusing on the conserved core of the protein. Tg–*T*. *gondii*; Sn–*S*. *neurona*; Pf–*P*. *falciparum*; At–*Arabidopsis thaliana;* Hs–human. Numbers on the right denote the residue numbers for the respective proteins.

To further explore the mechanism of inhibition of *S*. *neurona* by AN3661, parasites were treated with the chemical mutagen N-ethyl-N-nitrosourea (ENU) and grown in the presence of 90 nM AN3661, a concentration of the drug that was found to be highly effective for inhibiting growth of the parasite (Fig 1A). Seven drug-resistant single-cell clones were isolated, and five were successfully used for further analyses. Based on the prior studies indicating that CPSF73 is the target of AN3661, the coding region of this gene (SN3_01500330) was amplified by PCR, sequenced, and the sequences aligned with the wild-type *S*. *neurona* CPSF73 coding sequence. These comparisons revealed that all five clones carried a point mutation resulting in a Y668N change in the *S*. *neurona* protein (Fig 2), a position that is also altered in AN3661-resistant *T*. *gondii* and *P*. *falciparum* [7,8]. While it is possible that each clone may possess additional mutations, the consistent alteration of Y668 in five independent clones nonetheless supports the hypothesis that AN3661 targets CPSF73 in *S*. *neurona*, much as it does in other apicomplexans.

### Effects of AN3661 on gene expression and poly(A) site choice in *S*. *neurona*

To assess the effects of AN3661 on transcription dynamics of *S*. *neurona*, genome-wide transcription was assessed by preparing and sequencing 3’ end-directed cDNA tags (so-called PATSeq), and subsequent analyses. For this, bovine turbinate (BT) cell cultures were infected with *S*. *neurona* in triplicate and grown with or without 90 nM AN3661, a concentration of drug found to fully inhibit parasite growth (Fig 1A). Between 3 and 6 million reads were returned for each library (S1 File). After demultiplexing and trimming, the reads were mapped to the *S*. *neurona* SN3.E1 (GenBank accession GCA_000727475.1) and *Bos taurus* (Hereford) genomes (ARS-UCD1.2); the latter mapping was used to assess the effects of the inhibitor on transcriptional dynamics in the host cells (following section). To confirm the reproducibility of the libraries, global gene expression measurements were conducted and gene-by-gene comparisons or relative expression levels were made and presented as the Pearson correlation coefficients for each pairwise comparison. Values for these coefficients ranged from 0.96 to 0.99, with one exception (which yielded a coefficient of 0.85; S1 File).

**Fig 3:**
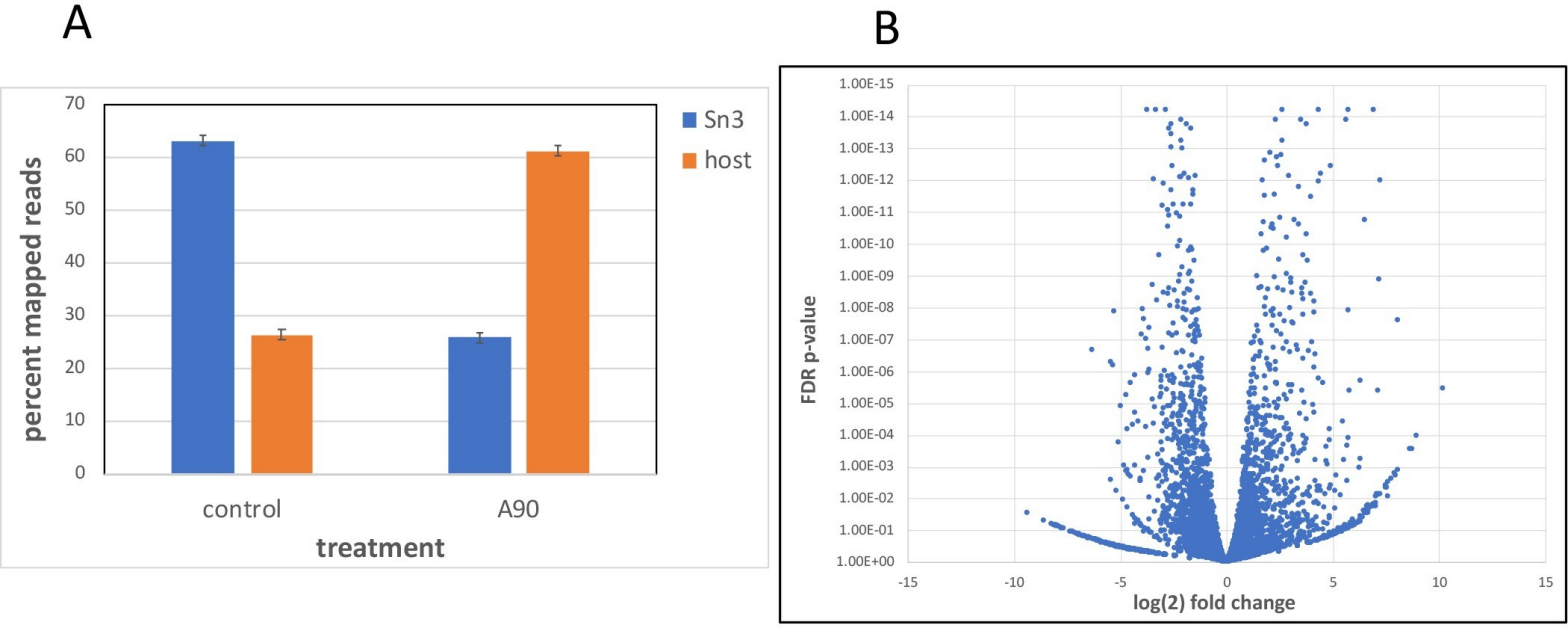
Gene expression in control and AN3661-treated cells. A. Overall parasite and host expression in control and AN3661-treated cells. The percent of all reads that map to the *S*. *neurona* and *B*. *taurus* genomes was calculated and plotted as shown. “control”–infected cells grown in the absence of AN3661. “A90”–infected cells treated with 90 nM AN3661. Reads mapping to the parasite genome are summarized with blue bars, and those mapping to the host cell genome with orange bars. Error bars denote standard deviations calculated from the values in each replicate of the respective sample. B. Volcano plot depicting changes in *S*. *neurona* gene expression due to AN3661 treatment.

To assess the overall effects of the CPSF73 inhibitor on transcriptional output, the relative quantities of reads that map to the two genomes were compared in control and AN3661-treated samples. As shown in Fig 3A, more than 60% of the reads mapped to the *S*. *neurona* genome in the non-treated cultures. In contrast, in AN3661-treated cultures, only about 25% of reads mapped to the parasite genome. This result indicates an almost 5-fold reduction in the overall proportion of transcripts from *S*. *neurona* that had been treated with the inhibitor, and is consistent with the growth characteristics of the parasite under these conditions (Fig 1A).

This analysis was augmented by a genome-wide analysis of parasite gene expression in control and inhibitor-treated cultures. As shown in Fig 3B and S3 File, in inhibitor-treated cells, 1253 genes showed a minimum of a 2-fold change in gene expression with false discovery rate adjusted p-values of 0.05 or less. This represents 18% of all annotated genes in the *S*. *neurona* SN3.E1 genome version used for this analysis, and 22% of all genes that showed some degree of gene expression. Genes encoding enzymes and proteins associated with protein biosynthesis were over-represented in the set of differentially-expressed genes (S3 File). This probably reflects a general effect of the inhibitor on transcriptional output and protein synthesis, and concomitant adjustments by the parasite.

Given the target of AN3661 (namely, CPSF73), these global transcriptomics analyses were supplemented with a study of the drug’s effects on poly(A) site choice in the parasite. For this, the analysis pipeline described earlier [16,17,19] was adapted to identify individual poly(A) sites whose relative usage was affected by the inhibitor. In this analysis, relative usage is defined as the fraction of all reads mapping to a given gene that also mapped to a given poly(A) site. This approach is biased towards identifying changes in sites whose usages may be low, and does not apply to genes that have only one poly(A) site. Nonetheless, it serves as a useful metric to assess effects on poly(A) site choice, the rationale being that changes that affect the core machinery will result in increased usage of minor, non-canonical poly(A) sites. The results of this analysis (S4 File) revealed that 1363 individual sites in 990 genes showed significant changes in poly(A) site usage using an false discovery rate (FDR)-adjusted p-value cutoff of 0.05. A more lenient cutoff (raw p-value < 0.05) yielded 5730 poly(A) sites in 2763 genes whose usage was altered in AN3661-treated cells. This is a substantial fraction of all genes (5955) with detectable levels of expression, and indicates a wide-ranging impact of the inhibitor on mRNA 3’ end formation.

**Fig 4:**
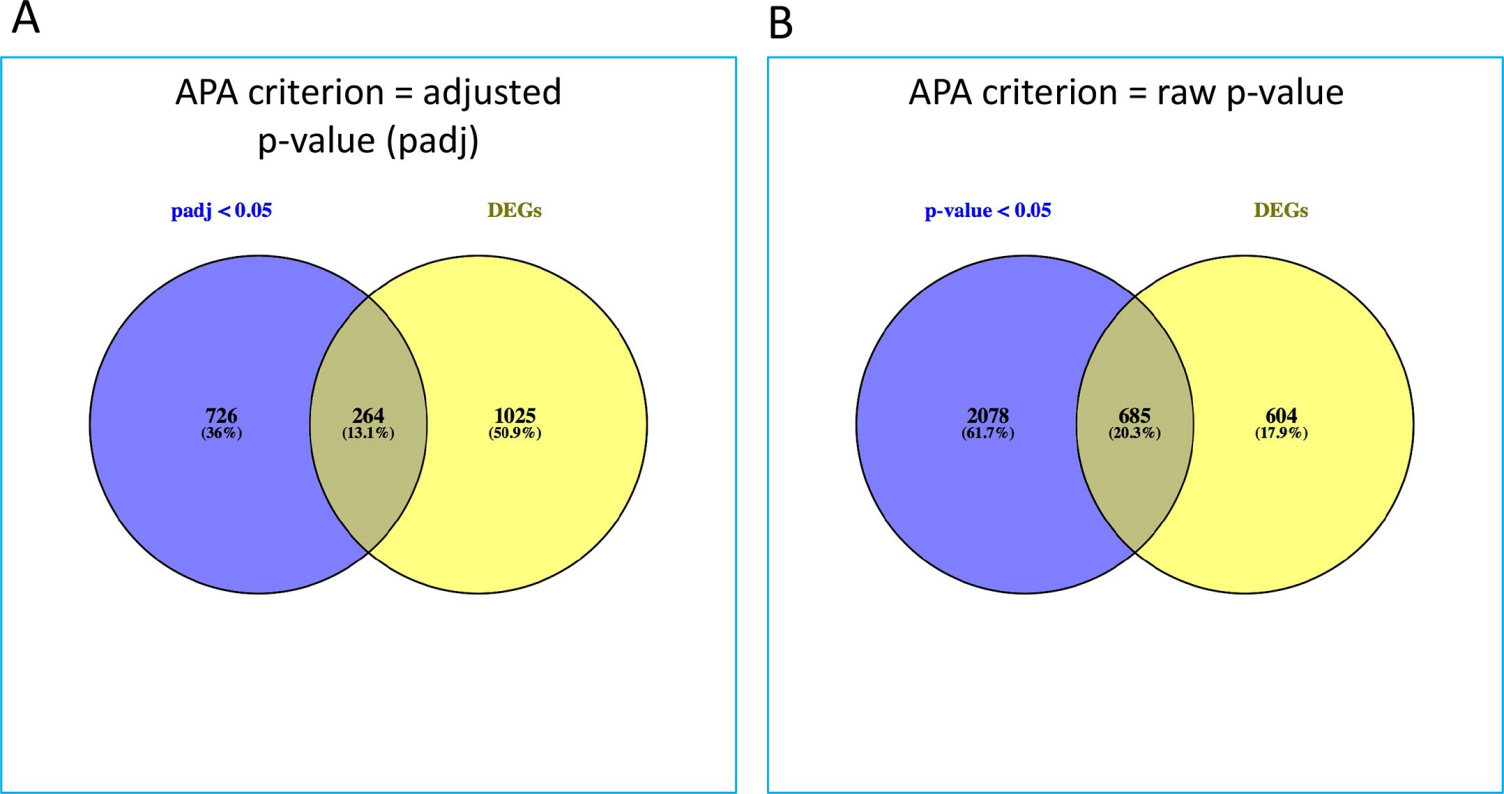
Genes that show differential poly(A) site usage and overall expression. Venn diagrams showing the overlaps between genes affected by APA (using the padj or p-value cutoffs described in the text, from S4 File; blue circles) and differentially expressed genes (from S3 File; yellow circles). Venn diagrams were made using Venny [20].

To assess the possible contribution of this altered poly(A) site choice to overall gene expression levels, the sets of genes whose expression change were compared with those affected by changes in poly(A) site choice. The results (Fig 4) show that between 24 and 27% of genes whose poly(A) site profiles change also showed significant changes in overall transcript levels. These results suggest that alternative polyadenylation (APA) may contribute to the overall pattern of gene expression in inhibitor-treated cells.

**Fig 5:**
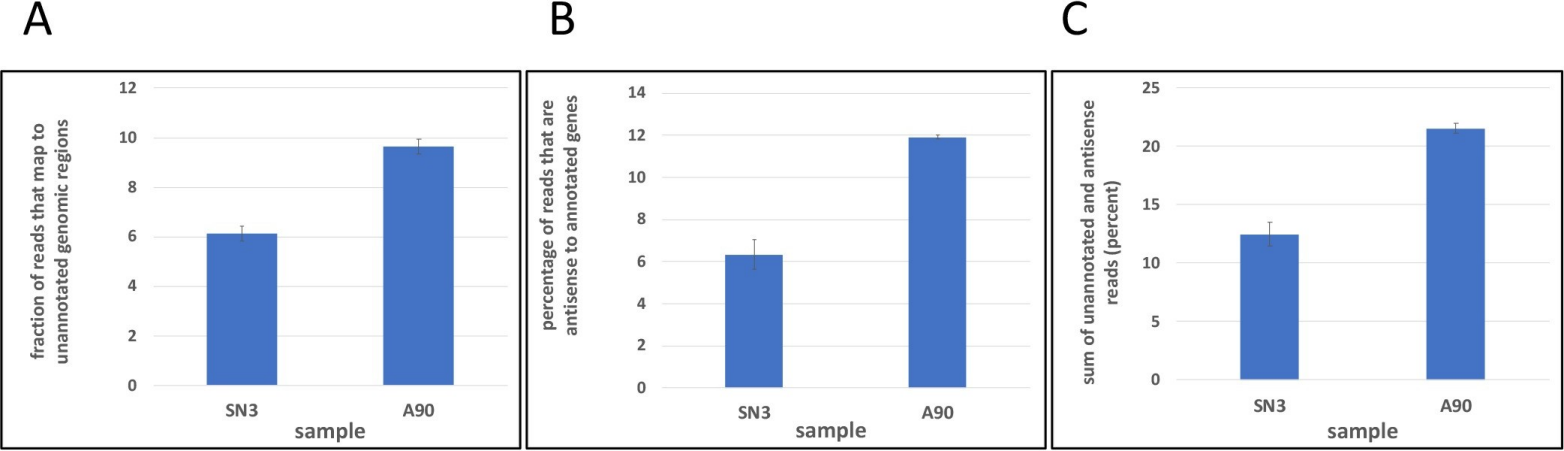
Fraction of all mapped reads that map outside of or antisense to annotated genes. Fraction (represented as percentages) of all mapped reads that map outside of or antisense to annotated genes. SN3 –untreated control cells. A90 –cells treated with 90 nM AN3661. Error bars denote standard deviations calculated from the values in each replicate of the respective sample. A. Reads that map outside of annotated regions. B. Reads that map antisense to annotated genes. C. Sum of data shown in panels A and B.

An inhibitor of mRNA polyadenylation is expected to alter transcription termination and increase the production of readthrough transcripts that might be “rescued” (and manifest as polyadenylated transcripts) through the use of cryptic or non-canonical polyadenylation sites. The latter would be apparent as an increased number of PATSeq reads mapping to unannotated parts of the genome or that are antisense in orientation to annotated genes. When these reads were measured, it was apparent that AN3661 increased by almost two-fold the overall levels of PATSeq reads that mapped outside of annotated regions (Fig 5A) or antisense to annotated genes (Fig 5B). The summed impact was that almost 10% of the total transcriptional output appeared to be associated with non-canonical poly(A) sites (Fig 5C). (Examples that illustrate these changes are shown in S1 Fig). This result corroborates those shown in S4 File and together support the hypothesis that AN3661 has a sizeable impact on transcription by altering mRNA polyadenylation.

### Effects of AN3661 on mRNA polyadenylation and gene expression in the BT host cells

**Fig 6:**
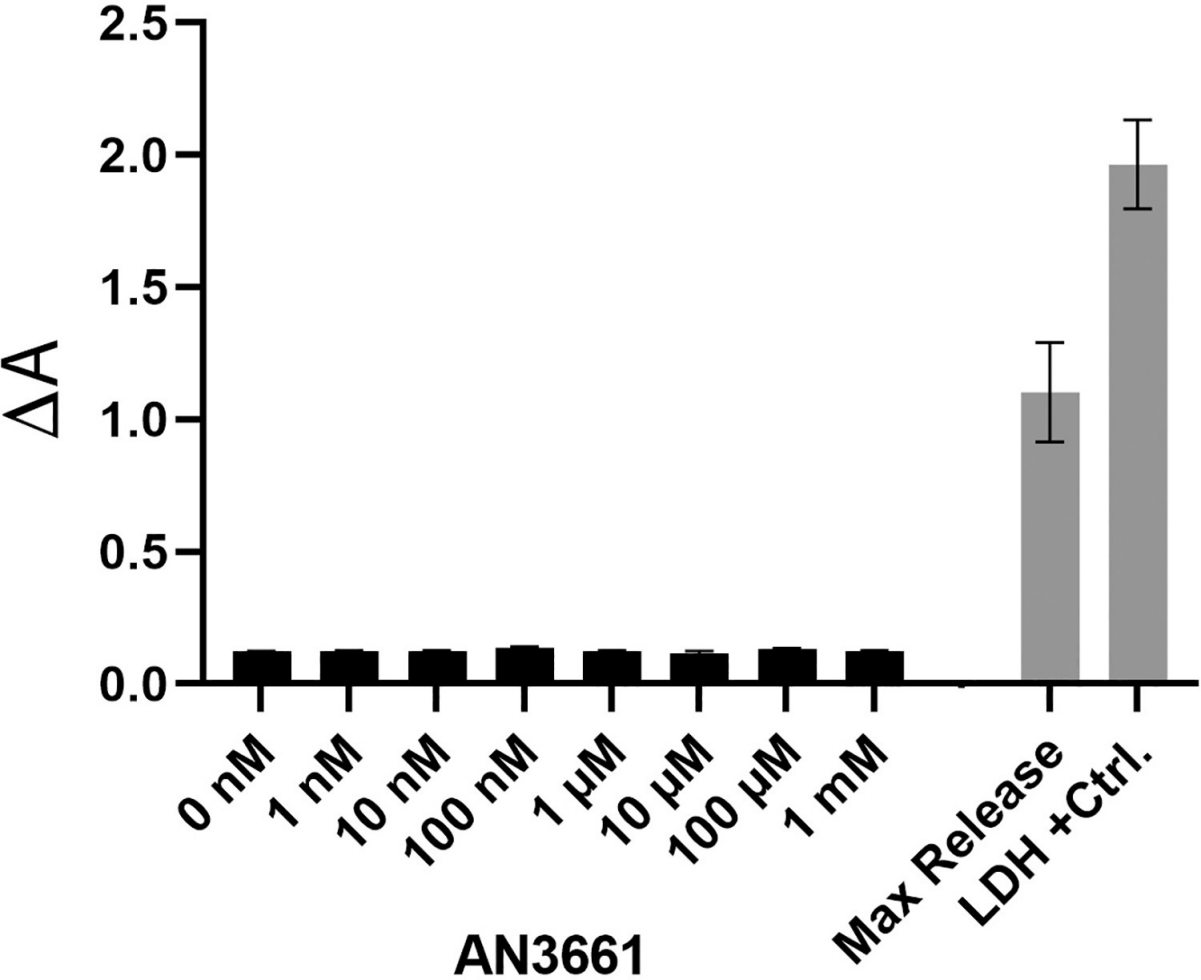
Cytotoxicity of AN3661 for bovine turbinate BT cells determined by LDH release. Triplicate wells of BT cultures in a 96-well plate were treated for 24 hours with drug concentrations from 1 nM to 1 mM, and the level of LDH released into the medium was compared to the LDH positive control provided by the manufacturer (Thermo Scientific), the maximum LDH release (i.e., lysed cells), and the spontaneous LDH release (non-treated cells). The LDH activity present in the samples was determined spectrophotometrically by subtracting the absorbance at 680 nm (background) from absorbance at 490 nm. Thus, ΔA = A490 nm−A_680 nm_, where A is absorbance.

In other systems, AN3661 has a high degree of specificity, with strong inhibition of the growth of *P*. *falciparum*, *T*. *gondii*, and *C*. *parvum* but little discernible effects on their respective hosts. To confirm that this is the case with the host cells used here for growth of *S*. *neurona*, cytotoxicity and gene expression was assessed in BT cells that were treated with AN3661. In a standard LDH release assay that measures cytotoxicity (Pierce LDH Cytotoxicity Assay), the LDH levels in media prepared from BT cells treated with AN3661 concentrations ranging from 1 nM to 1 mM were indistinguishable from LDH released from non-treated cells (0 nM), and were only slightly more than 10% of the maximum release (lysed cells) control (Fig 6). Therefore, AN3661 was not cytotoxic to the BT host cell line, even at concentrations approaching 100,000-fold greater than the IC_50_ for *S*. *neurona* (14.99 nM; Fig 1B).

To assess the effects of AN3661 on gene expression in the BT host cells, PATSeq libraries were prepared from uninfected cells grown in the absence or presence of 90 nM AN3661 (the same concentration as was used to study gene expression in *S*. *neurona*-infected cells). These libraries were sequenced using the Illumina platform and mapped to the *B*. *taurus* (Hereford) genome. Consistency between replicates was assessed by comparisons of genome-wide expression in individual replicates. These pairwise comparisons of libraries prepared from uninfected host cells yielded consistently high Pearson Correlation coefficients (uniformly greater than 0.96; S1 File). Given this, several additional analyses were conducted to assess the impacts of AN3661 on gene expression and polyadenylation.

**Fig 7:**
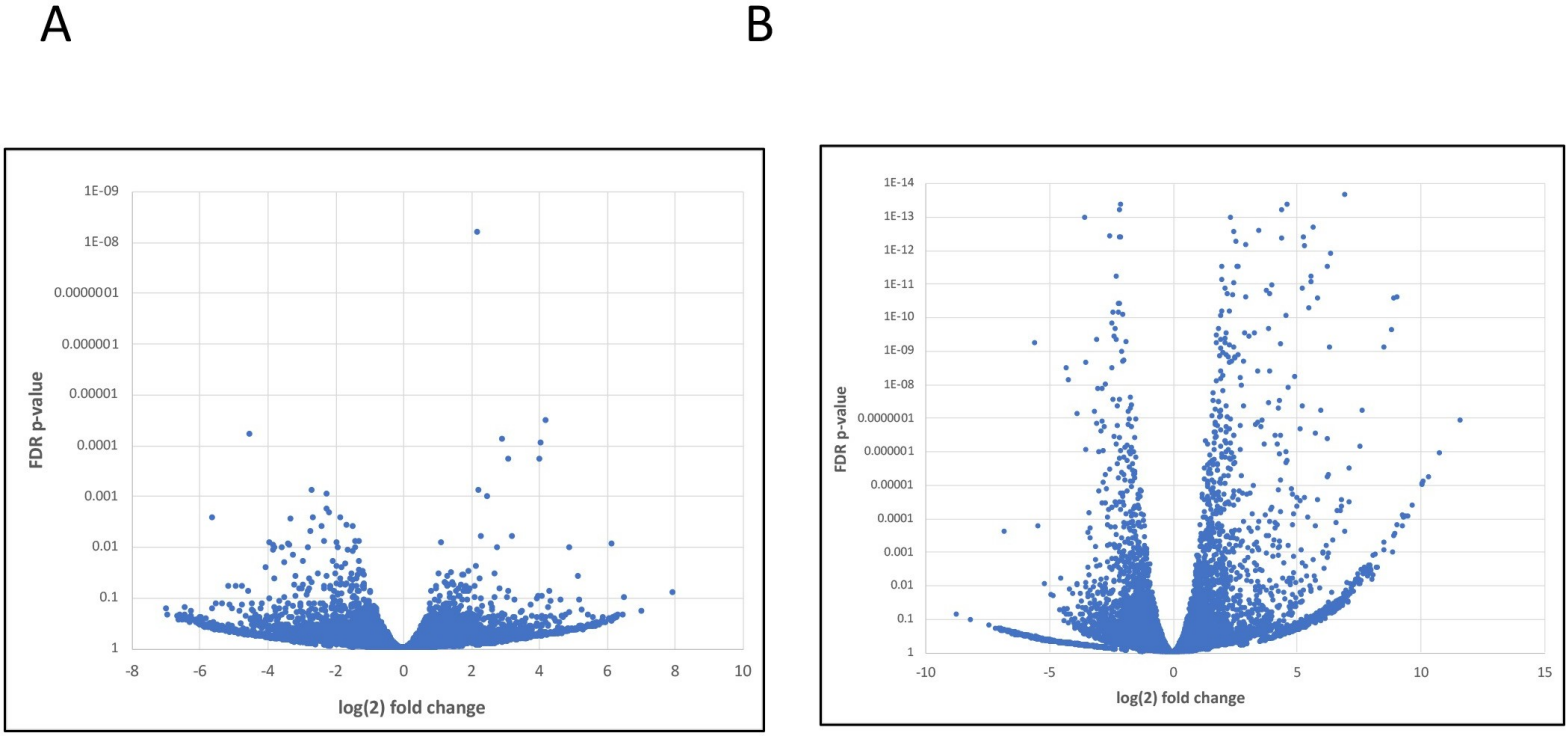
Volcano plots showing the extent of differential host gene expression in infected and AN3661-treated cells. A. Comparison of host cell gene expression in untreated and AN3661-treated cells. B. Comparison of host cell gene expression in uninfected and *S*. *neurona*-infected cells.

As was done for the analysis of *S*. *neurona* gene expression, gene-by-gene expression in treated and control host cells was measured. The results showed that only 78 genes (of 14,785 whose expression could be measured in this study) had significantly-different expression (2-fold or greater change in expression, with an FDR-adjusted p-value less than 0.05) in AN3661-treated BT host cells (Fig 7A, S5 File). By way of comparison, *S*. *neurona* incited changes in the expression of 1489 genes in the host cells (Fig 7B, S6 File). Together, these results indicate that AN3661 has a minimal impact on overall gene expression in BT host cells.

In parallel, the effect of AN3661 on poly(A) site choice in uninfected BT cells was assessed using the analysis pipeline described in S2 File. A site-by-site analysis of poly(A) site usage in control and treated cells showed that none of the >35,000 sites for which results could be returned showed changes that satisfied the FDR-adjusted p-value cut-off of 0.05 (S7 File). Using the more lenient raw p-value cutoff of 0.05 (as was done for the analysis of *S*. *neurona* poly(A) site choice described above), 32 sites from 31 genes were returned. These results indicate minimal effect of AN3661 on mRNA polyadenylation in BT cells.

**Fig 8:**
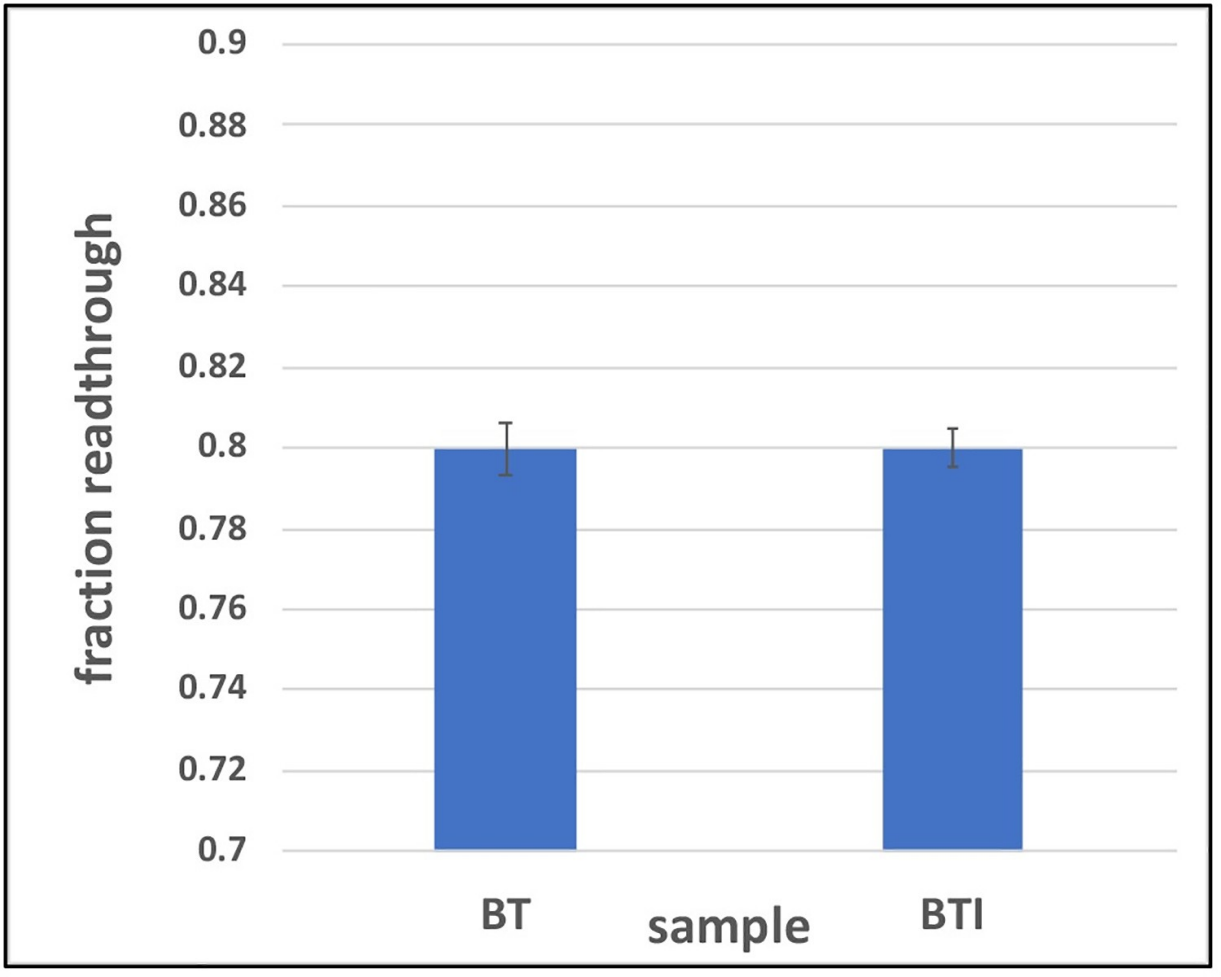
Transcriptional readthrough in BT host cells. Fraction of all mapped reads that map outside of or antisense to annotated genes in control and AN3661-treated host cells was measured and plotted as shown. BT–untreated control cells. BTI–cells treated with 90 nM AN3661. For this plot, the fractions of reads that map outside of and antisense to annotated genes were summed and plotted as shown. Note the scale of the y-axis.

To further explore possible effects on transcription, the numbers of PATSeq reads that map outside of, or antisense to, annotated BT genes was tabulated. The result of this exercise showed that there were no appreciable differences in the quantities of such reads between control and treated BT cells (Fig 8). Together, these results indicate that AN3661 has almost no impact on gene expression, mRNA polyadenylation, and transcriptional readthrough in BT cells.

## Discussion

### AN3661 targets CPSF73 and mRNA polyadenylation in *S*. *neurona*

In recent years, a variety of benzoxaboroles have been found to target mRNA processing in a number of unicellular parasites, including several that can cause disease in humans [7–9,21–23]. The benzoxaborole AN3661, the subject of this study, inhibits *P*. *falciparum*, *T*. *gondii*, *and C*. *parvum* by targeting CPSF73, a subunit of the parasite polyadenylation complex [7–9]. The results presented in this study extend the range of apicomplexan parasites that are inhibited by AN3661 to include two species that have importance in agriculture. Moreover, the finding that AN3661-resistant *S*. *neurona* clones carry mutations in the gene that encodes CPSF73 corroborates earlier studies indicating that this drug targets the mRNA polyadenylation in apicomplexan parasites.

While the target of AN3661 and the physiological consequences of treatment with the drug have been well-established [7–9], a detailed understanding of the consequences of AN3661 treatment on genome-wide transcription dynamics in apicomplexans parasites is lacking. The results presented in this report provide added insight into the effects of the drug on 3’ end processing and transcription termination. Treatment with the drug results in a general diminution of parasite transcript levels in infected cells (Fig 3A). This is consistent with the growth inhibition attendant with drug treatment (Fig 1). Interestingly, some genes are more affected by drug treatment than others, resulting in a wide-ranging change in relative gene expression in the two conditions (Fig 3B). These changes in all likelihood reflect the differential responses of gene expression to the physiological changes brought about by the alterations in mRNA polyadenylation incited by the drug.

Along with the overall transcript-level response, treatment with AN3661 also leads to a global alteration in poly(A) site choice in *S*. *neurona*, affecting almost half of all expressed genes in the parasite. This is very likely a consequence of a general inhibition of 3’ end processing by the drug. AN3661 binds in the active site of the apicomplexan CPSF73 in a manner predicted to interfere with the metal ion-mediated catalytic mechanism of the endonuclease [9]. Inhibition of processing at the primary (preferred) poly(A) site is expected to result in a population of extended primary transcripts, the collection of which would present numerous alternate poly(A) signals to the processing apparatus. Any of these sites might be recognized and processed, guided by the dynamics of the interactions between the nascent RNA and the poly(A) complex. Weak sites that are usually skipped might be utilized at greater frequencies in drug-treated cells. There is increased read-through transcription in drug-treated *S*. *neurona* cells (Fig 5), and additional potential sites might be present in extended transcripts. Taken together, the global shifts in poly(A) site choice upon AN3661 treatment are consistent with the reported mode of action of the drug [7–9].

In these regards, the effects of AN3661 on polyadenylation in *S*. *neurona* are similar to the consequences of reduction of CPSF73 levels or activity in mammalian cells and in yeast. The mammalian CPSF73 is a target of a compound (JTE-607) that is active against acute myeloid leukemia and Ewings sarcoma cell lines, and acts by mimicking RNA binding at the CPSF73 active site [24,25]. In JTE-607 treated cells, a substantial increase in read-through transcription is seen [25], much as is seen in AN3661-treated *S*. *neurona* cells (Fig 5). This parallel is supportive of the model for the proposed mechanism of action of AN3661 in *S*. *neurona*, namely that the compound inhibits CPSF73 and alters efficient mRNA polyadenylation and transcription termination.

YSH1 is the yeast counterpart of CPSF73 and resides in a complex that includes other CPSF subunit orthologs [26]. Like the mammalian CPSF73, YSH1 is the endonuclease that cleaves the pre-mRNA prior to polyadenylation. YSH1 levels are maintained in part by the action of the *IPA1* gene product, such that *ipa1* mutants have substantially-reduced levels of YSH1 [27]. *ipa1* mutants exhibit substantial degrees of transcriptional readthrough, and also considerable changes in the usage of annotated poly(A) sites [28]. These are features also seen in AN3661-treated *S*. *neurona* cells. This parallel provides further support for the proposed mechanism of action of AN3661 in *S*. *neurona*.

### AN3661 has a minimal impact on host cell transcription dynamics

A remarkable feature of AN3661 is its selectivity as far as cellular toxicity is concerned. Previously, it was shown that AN3661 had little effect on the growth of cells or animals that serve as hosts for *P*. *falciparum*, *T*. *gondii*, and *C*. *parvum* [7–9]. The results presented in this study show that the drug does not affect the growth of the BT cells used for propagation of *S*. *neurona*. This reinforces the theme that AN3661 has high specificity for its target in the parasites, and does not affect the host (mammalian) ortholog, CPSF73.

This specificity implies that the drug has no impact on mRNA polyadenylation in host cells or animals. However, the compound JTE-607 (mentioned above) is an inhibitor of CPSF73 function that selectively inhibits the growth of acute myeloid leukemia and Ewing’s sarcoma cell lines [24,25], and has been shown to prolong life in a mouse leukemia model [29]. This compound causes wide-ranging changes in poly(A) site usage and transcriptional readthrough in mammalian cells [25]. That a selective compound can alter mRNA polyadenylation raises the possibility that AN3661 may alter 3’ end processing, but in ways that have minimal or no impact on host cell physiology. It is thus important that AN3661 had no discernible effect on mRNA polyadenylation and transcription dynamics in BT host cells. In particular, there was no discernible impact of the drug on poly(A) site usage in BT cells (S7 File). Moreover, there were no indications of increased usage of distal poly(A) sites in AN3661-treated BT cells (Fig 8), nor was there evidence for the usage of novel sites downstream of annotated transcription units (S7 File). These parameters (altered poly(A) site choice, increased distal poly(A) site usage, and transcription downstream of annotated genes) are hallmarks of the inhibition of CPSF73 in mammals and yeast [24,25,27,28]. That AN3661 does not alter these features of gene expression in BT cells is strong evidence that the compound in fact does not interfere with CPSF73 functioning in these cells.

The results presented in this report strengthen the case for AN3661 (and related compounds that also target CPSF73 in parasites) as therapeutic agents. However, enthusiasm for such a use is tempered by the scope of mutations in the parasite CPSF73 that can reduce sensitivity to the drug. In *S*. *neurona*, single mutations near the CPSF73 active site were sufficient to provide resistance to the compound (Fig 2). Resistance also could be achieved by single base changes in *P*. *falciparum* [7] and *T*. *gondii* [8]. The reported frequency of spontaneous resistance clones *in vitro* in *P*. *falciparum* (with resistant clones arising readily in initial populations of as few as 10^6^ cells) [7] is especially notable, as this frequency indicates that resistance would likely arise quickly during a treatment regimen. However, when used in concert with drugs that target different processes, compounds that selectively target CPSF73 in apicomplexans may find use. The absence of effects of the drug on host cells makes such uses attractive, as AN3661 would likely not add any side effects when used in a combination therapy.

## Conclusions

The results reported in this study show that the benzoxaborole AN3661 inhibits the growth of *S*. *neurona*, an apicomplexan parasite of horses and marine mammals. This inhibition can be attributed to the targeting by the drug of CPSF73. AN3661 had a minimal impact on the bovine host cells used to propagate the parasites; importantly, there was no discernible effect of the drug on poly(A) site choice or overall gene expression in the host cells. Collectively, these results reveal AN3661 to be an exceedingly selective inhibitor of apicomplexan mRNA polyadenylation.

## Supporting information

S1 Fig Changes in transcriptional dynamics in *S*. neurona treated with AN3661. Browser tracks showing examples of changes in poly(A) site usage in AN3661-treated cells. The order for each representation is (top to bottom): chromosomal location (in bp), gene annotation, coding region (CDS) annotation, reads from un-treated cells (Sn3 mapping), and reads from AN3661-treated cells (A90-1 mapping). Reads colored green are oriented in the sense (5’->3’ left to right) direction, and reads colored green are oriented in the antisense direction. Tracks were created using CLC Genomics Workbench.
(PDF)

S1 Table Primers used in this study. (XLSX)

S1 File Reads and mapping statistics. (XLSX)

S2 File Analysis pipeline for poly(A) site determinations. (DOCX)

S3 File Gene expression analysis in AN3661-treated *S*. *neurona*. *S*. *neurona*-infected BT cells were treated with 90 nM AN3661, RNA isolated, and PATSeq libraries constructed and sequenced. PATSeq reads were used to assess gene expression in drug-treated *S*. *neurona* cells.
(XLSX)

S4 File Poly(A) site analysis in AN3661-treated *S*. *neurona*. *S*. *neurona*-infected BT cells were treated with 90 nM AN3661, RNA isolated, and PATSeq libraries constructed and sequenced. The data were used to assess poly(A) site choice in the parasite in the two conditions.
(XLSX)

S5 File Gene expression in control and AN3661-treated BT cells. RNA was isolated from control and AN3661-treated BT cells and PATSeq libraries constructed and sequenced. PATSeq reads were used to assess host gene expression.
(XLSX)

S6 File Host gene expression in uninfected and *S*. *neurona*- infected BT cells. RNA was isolated from control and *S*. *neurona*-infected BT cells and PATSeq libraries constructed and sequenced. PATSeq reads were used to assess host gene expression.
(XLSX)

S7 File Poly(A) site analysis in AN3661-treated BT host cells. RNA was isolated from control and AN3661-treated BT cells and PATSeq libraries constructed and sequenced. The data were used to assess poly(A) site choice in the host in the two conditions.
(XLSX)

## References

[1] Levine ND. The protozoan phylum Apicomplexa: CRC Press; 1988. doi: 10.1201/9781351076111

[2] Reichel MP, Alejandra Ayanegui-Alcerreca M, Gondim LF, Ellis JT. What is the global economic impact of Neospora caninum in cattle—the billion dollar question. Int J Parasitol. 2013;43(2):133–42. Epub 2012/12/19. doi: 10.1016/j.ijpara.2012.10.022 . 23246675

[3] Chapman HD, Barta JR, Blake D, Gruber A, Jenkins M, Smith NC, et al. A selective review of advances in coccidiosis research. Adv Parasitol. 2013;83:93–171. doi: 10.1016/B978-0-12-407705-8.00002-1 . 23876872

[4] Dubey JP, Hemphill A, Calero-Bernal R, Schares G. Neosporosis in Animals: CRC Press; 2017. 529 p.

[5] Dubey JP, Howe DK, Furr M, Saville WJ, Marsh AE, Reed SM, et al. An update on *Sarcocystis neurona* infections in animals and equine protozoal myeloencephalitis (EPM). Vet Parasitol. 2015;209(1–2):1–42. doi: 10.1016/j.vetpar.2015.01.026 ; PubMed Central PMCID: PMC4461864. 25737052 PMC4461864

[6] Chapman HD, Jeffers TK, Williams RB. Forty years of monensin for the control of coccidiosis in poultry. Poult Sci. 2010;89(9):1788–801. doi: 10.3382/ps.2010-00931 . 20709963

[7] Sonoiki E, Ng CL, Lee MC, Guo D, Zhang YK, Zhou Y, et al. A potent antimalarial benzoxaborole targets a Plasmodium falciparum cleavage and polyadenylation specificity factor homologue. Nat Commun. 2017;8:14574. doi: 10.1038/ncomms14574 ; PubMed Central PMCID: PMC5343452. 28262680 PMC5343452

[8] Palencia A, Bougdour A, Brenier-Pinchart MP, Touquet B, Bertini RL, Sensi C, et al. Targeting Toxoplasma gondii CPSF3 as a new approach to control toxoplasmosis. EMBO Mol Med. 2017;9(3):385–94. doi: 10.15252/emmm.201607370 ; PubMed Central PMCID: PMC5331205. 28148555 PMC5331205

[9] Swale C, Bougdour A, Gnahoui-David A, Tottey J, Georgeault S, Laurent F, et al. Metal-captured inhibition of pre-mRNA processing activity by CPSF3 controls Cryptosporidium infection. Sci Transl Med. 2019;11(517). Epub 2019/11/07. doi: 10.1126/scitranslmed.aax7161 . 31694928

[10] Howe DK, Gaji RY, Mroz-Barrett M, Gubbels MJ, Striepen B, Stamper S. *Sarcocystis neurona* merozoites express a family of immunogenic surface antigens that are orthologues of the *Toxoplasma gondii* surface antigens (SAGs) and SAG-related sequences. Infect Immun. 2005;73(2):1023–33. doi: 10.1128/IAI.73.2.1023-1033.2005 15664946 PMC546929

[11] Gaji RY, Zhang D, Breathnach CC, Vaishnava S, Striepen B, Howe DK. Molecular genetic transfection of the coccidian parasite *Sarcocystis neurona*. Mol Biochem Parasitol. 2006;150(1):1–9. doi: 10.1016/j.molbiopara.2006.06.003 . 16844242

[12] Pfefferkorn ER, Pfefferkorn LC. *Toxoplasma gondii*: Characterization of a mutant resistant to 5-fluorodeoxyuridine. Experimental Parasitology. 1977;42:44–55. doi: 10.1016/0014-4894(77)90060-1 140809

[13] Howe DK, Yeargan M, Simpson L, Dangoudoubiyam S. Molecular Genetic Manipulation of *Sarcocystis neurona*. Curr Protoc Microbiol. 2018;48:20D 2 1–D 2 14. doi: 10.1002/cpmc.48 . 29512112

[14] Ma L, Pati PK, Liu M, Li QQ, Hunt AG. High throughput characterizations of poly(A) site choice in plants. Methods. 2014;67(1):74–83. Epub 2013/07/16. doi: 10.1016/j.ymeth.2013.06.037 ; PubMed Central PMCID: PMC3900603. 23851255 PMC3900603

[15] Pati PK, Ma L, Hunt AG. Genome-wide determination of poly(A) site choice in plants. Methods Mol Biol. 2015;1255:159–74. Epub 2014/12/10. doi: 10.1007/978-1-4939-2175-1_14 . 25487212

[16] Bell SA, Shen C, Brown A, Hunt AG. Experimental Genome-Wide Determination of RNA Polyadenylation in Chlamydomonas reinhardtii. PLoS One. 2016;11(1):e0146107. Epub 2016/01/06. doi: 10.1371/journal.pone.0146107 ; PubMed Central PMCID: PMC4701671. 26730730 PMC4701671

[17] de Lorenzo L, Sorenson R, Bailey-Serres J, Hunt AG. Noncanonical Alternative Polyadenylation Contributes to Gene Regulation in Response to Hypoxia. Plant Cell. 2017;29(6):1262–77. Epub 2017/06/01. doi: 10.1105/tpc.16.00746 ; PubMed Central PMCID: PMC5502444. 28559476 PMC5502444

[18] Ojo KK, Dangoudoubiyam S, Verma SK, Scheele S, DeRocher AE, Yeargan M, et al. Selective inhibition of *Sarcocystis neurona* calcium-dependent protein kinase 1 for equine protozoal myeloencephalitis therapy. Int J Parasitol. 2016;46(13–14):871–80. doi: 10.1016/j.ijpara.2016.08.003 ; PubMed Central PMCID: PMC5130624. 27729271 PMC5130624

[19] Stevens AT, Howe DK, Hunt AG. Characterization of mRNA polyadenylation in the apicomplexa. PLoS One. 2018;13(8):e0203317. Epub 2018/08/31. doi: 10.1371/journal.pone.0203317 . 30161237 PMC6117058

[20] Oliveros JC. Venny. An interactive tool for comparing lists with Venn’s diagrams. 2007–2015 [cited 2020 December 31]. Available from: https://bioinfogp.cnb.csic.es/tools/venny/index.html.

[21] Begolo D, Vincent IM, Giordani F, Pohner I, Witty MJ, Rowan TG, et al. The trypanocidal benzoxaborole AN7973 inhibits trypanosome mRNA processing. PLoS Pathog. 2018;14(9):e1007315. Epub 2018/09/27. doi: 10.1371/journal.ppat.1007315 ; PubMed Central PMCID: PMC6173450 following competing interests: Yvonne Freund was employed by Anacor Pharmaceuticals. Benzoxaboroles were supplied by Anacor Pharmaceuticals (YF) which was later taken over by Pfizer. This does not alter our adherence to all the PLOS Pathogens policies on sharing data and materials. 30252911 PMC6173450

[22] Bellini V, Swale C, Brenier-Pinchart MP, Pezier T, Georgeault S, Laurent F, et al. Target Identification of an Antimalarial Oxaborole Identifies AN13762 as an Alternative Chemotype for Targeting CPSF3 in Apicomplexan Parasites. iScience. 2020;23(12):101871. Epub 2020/12/19. doi: 10.1016/j.isci.2020.101871 ; PubMed Central PMCID: PMC7733022. 33336164 PMC7733022

[23] Wall RJ, Rico E, Lukac I, Zuccotto F, Elg S, Gilbert IH, et al. Clinical and veterinary trypanocidal benzoxaboroles target CPSF3. Proc Natl Acad Sci U S A. 2018;115(38):9616–21. Epub 2018/09/07. doi: 10.1073/pnas.1807915115 ; PubMed Central PMCID: PMC6156652. 30185555 PMC6156652

[24] Kakegawa J, Sakane N, Suzuki K, Yoshida T. JTE-607, a multiple cytokine production inhibitor, targets CPSF3 and inhibits pre-mRNA processing. Biochem Biophys Res Commun. 2019;518(1):32–7. Epub 2019/08/11. doi: 10.1016/j.bbrc.2019.08.004 . 31399191

[25] Ross NT, Lohmann F, Carbonneau S, Fazal A, Weihofen WA, Gleim S, et al. CPSF3-dependent pre-mRNA processing as a druggable node in AML and Ewing’s sarcoma. Nat Chem Biol. 2020;16(1):50–9. Epub 2019/12/11. doi: 10.1038/s41589-019-0424-1 ; PubMed Central PMCID: PMC7116157. 31819276 PMC7116157

[26] Hill CH, Boreikaite V, Kumar A, Casanal A, Kubik P, Degliesposti G, et al. Activation of the Endonuclease that Defines mRNA 3’ Ends Requires Incorporation into an 8-Subunit Core Cleavage and Polyadenylation Factor Complex. Mol Cell. 2019;73(6):1217–31 e11. Epub 2019/02/10. doi: 10.1016/j.molcel.2018.12.023 ; PubMed Central PMCID: PMC6436931. 30737185 PMC6436931

[27] Pearson EL, Graber JH, Lee SD, Naggert KS, Moore CL. Ipa1 Is an RNA Polymerase II Elongation Factor that Facilitates Termination by Maintaining Levels of the Poly(A) Site Endonuclease Ysh1. Cell Rep. 2020;32(5):108031. Epub 2020/08/07. doi: 10.1016/j.celrep.2020.108031 . 32755571

[28] Pearson EL, Graber JH, Lee SD, Naggert KS, Moore CL. Ipa1 Is an RNA Polymerase II Elongation Factor that Facilitates Termination by Maintaining Levels of the Poly(A) Site Endonuclease Ysh1. Cell Rep. 2019;26(7):1919–33 e5. Epub 2019/02/14. doi: 10.1016/j.celrep.2019.01.051 ; PubMed Central PMCID: PMC7236606. 30759400 PMC7236606

[29] Tajima N, Fukui K, Uesato N, Maruhashi J, Yoshida T, Watanabe Y, et al. JTE-607, a multiple cytokine production inhibitor, induces apoptosis accompanied by an increase in p21waf1/cip1 in acute myelogenous leukemia cells. Cancer Sci. 2010;101(3):774–81. Epub 2009/12/24. doi: 10.1111/j.1349-7006.2009.01446.x . 20028380 PMC11159651

