# Supplementary material for: Transcriptional dynamics in the protozoan parasite Sarcocystis neurona and mammalian host cells after treatment with a specific inhibitor of apicomplexan mRNA polyadenylation
Source: PLoS One. 2021 Oct 28;16(10):e0259109. doi: 10.1371/journal.pone.0259109 (PMC8553156; doi:10.1371/journal.pone.0259109)
Supplement: S1 Fig — Browser tracks showing examples of changes in poly(A) site usage in AN3661-treated cells. The order for each representation is (top to bottom): chromosomal location (in bp), gene annotation, coding region (CDS) annotation, reads from un-treated cells (Sn3 mapping), and reads from AN3661-treated cells (A90-1 mapping). Reads colored green are oriented in the sense (5’->3’ left to right) direction, and reads colored green are oriented in the antisense direction. Tracks were created using CLC Genomics Workbench. (PDF) [file pone.0259109.s001.pdf]

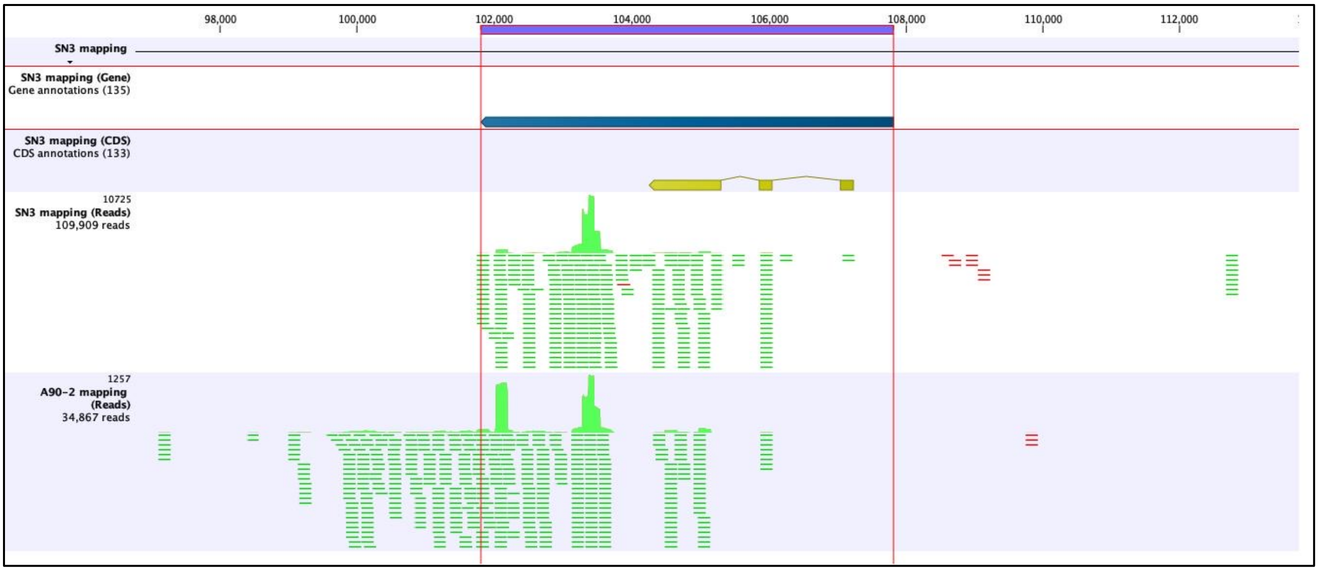
98,000
100,000
102,000
104,000
106,000
108,000
110,000
112,000
SN3 mapping
SN3 mapping (Gene)
Gene annotations (135)
SN3 mapping (CDS)
CDS annotations (133)
10725
SN3 mapping (Reads)
109,909 reads
1257
A90-2 mapping (Reads)
34,867 reads

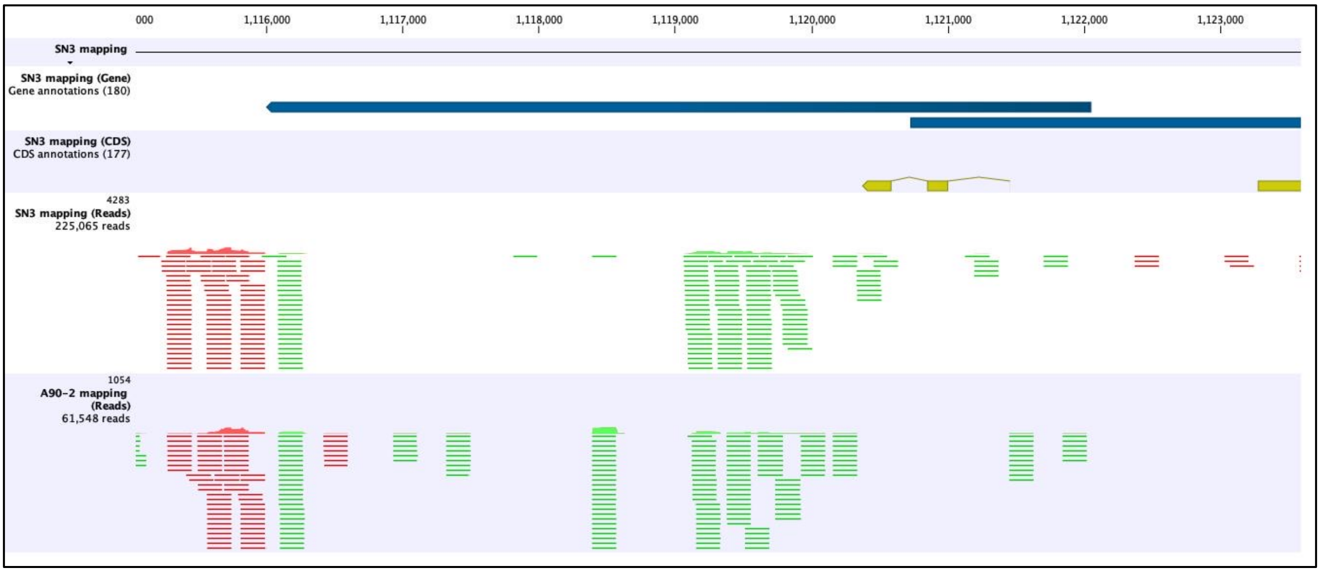
000
1,116,000
1,117,000
1,118,000
1,119,000
1,120,000
1,121,000
1,122,000
1,123,000
SN3 mapping
SN3 mapping (Gene)
Gene annotations (180)
SN3 mapping (CDS)
CDS annotations (177)
4283
SN3 mapping (Reads)
225,065 reads
1054
A90-2 mapping (Reads)
61,548 reads

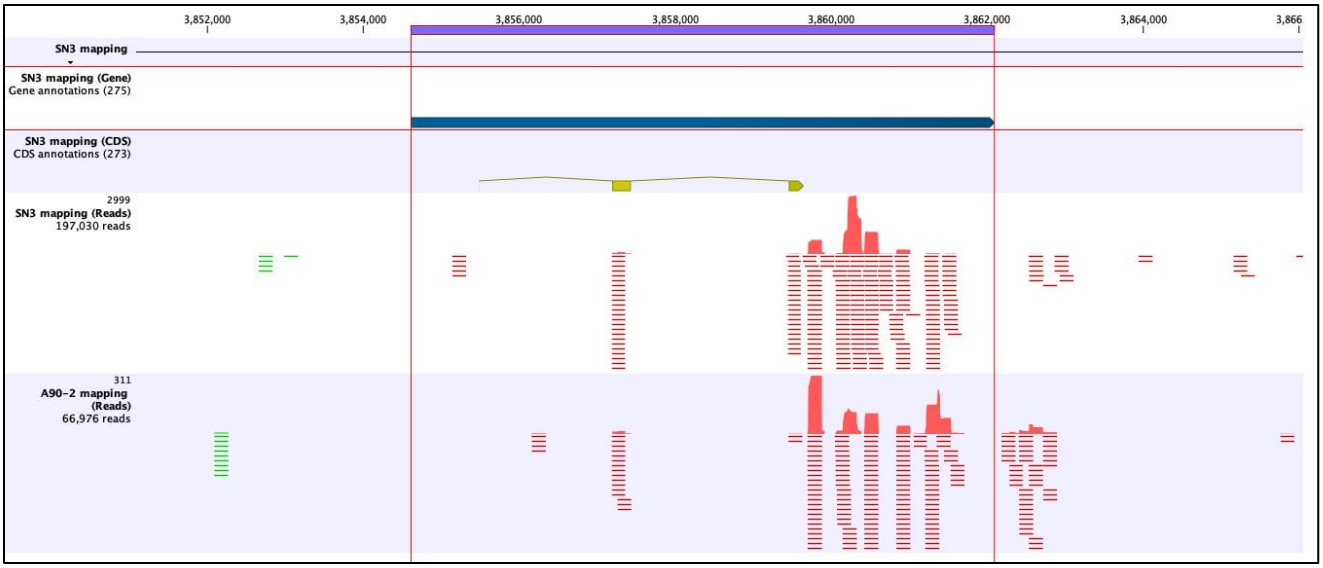
3,852,000
3,854,000
3,856,000
3,858,000
3,860,000
3,862,000
3,864,000
3,866
SN3 mapping
SN3 mapping (Gene)
Gene annotations (275)
SN3 mapping (CDS)
CDS annotations (273)
2999
SN3 mapping (Reads)
197,030 reads
311
A90-2 mapping (Reads)
66,976 reads

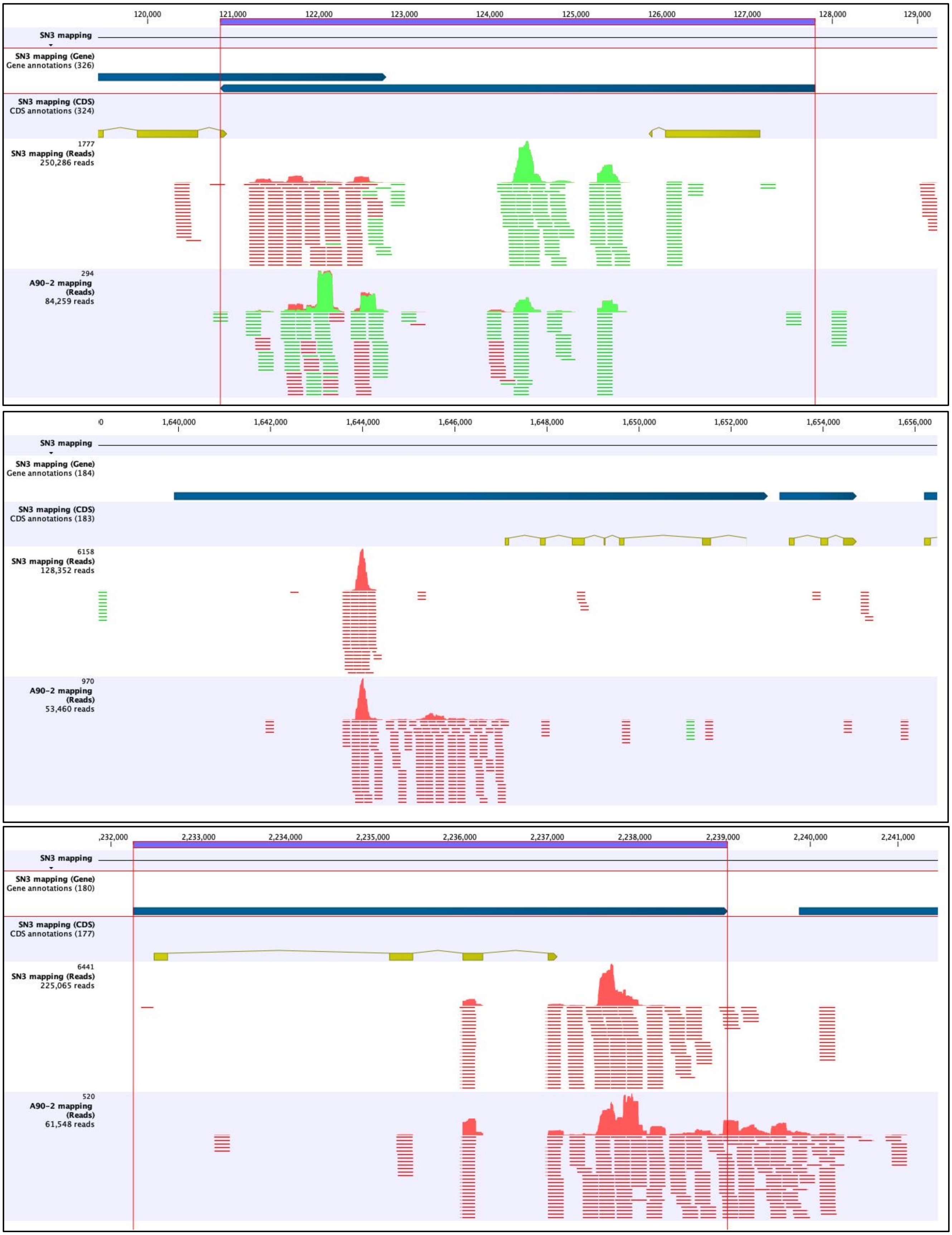
120,000
121,000
122,000
123,000
124,000
125,000
126,000
127,000
128,000
129,000
SN3 mapping
SN3 mapping (Gene)
Gene annotations (326)
SN3 mapping (CDS)
CDS annotations (324)
1777
SN3 mapping (Reads)
250,286 reads
294
A90-2 mapping
(Reads)
84,259 reads
1,640,000
1,642,000
1,644,000
1,646,000
1,648,000
1,650,000
1,652,000
1,654,000
1,656,000
SN3 mapping
SN3 mapping (Gene)
Gene annotations (184)
SN3 mapping (CDS)
CDS annotations (183)
6158
SN3 mapping (Reads)
128,352 reads
970
A90-2 mapping
(Reads)
53,460 reads
2,233,000
2,234,000
2,235,000
2,236,000
2,237,000
2,238,000
2,239,000
2,240,000
2,241,000
SN3 mapping
SN3 mapping (Gene)
Gene annotations (180)
SN3 mapping (CDS)
CDS annotations (177)
6441
SN3 mapping (Reads)
225,065 reads
520
A90-2 mapping
(Reads)
61,548 reads

Supplemental Figure 1. Browser tracks showing examples of changes in poly(A) site usage in AN3661-treated cells. The order for each representation is (top to bottom): chromosomal location (in bp), gene annotation, coding region (CDS) annotation, reads from un-treated cells (Sn3 mapping), and reads from AN3661-treated cells (A90-1 mapping). Reads colored green are oriented in the sense (5'->3' left to right) direction, and reads colored green are oriented in the antisense direction. Tracks were created using CLC Genomics Workbench.
