# Supplementary material for: Transcriptional dynamics in the protozoan parasite Sarcocystis neurona and mammalian host cells after treatment with a specific inhibitor of apicomplexan mRNA polyadenylation
Source: PLoS One. 2021 Oct 28;16(10):e0259109. doi: 10.1371/journal.pone.0259109 (PMC8553156; doi:10.1371/journal.pone.0259109)
Supplement: S2 File — (DOCX) [file pone.0259109.s004.docx]

**Supplemental File 2**

**Section I. Poly(A) Tag (PAT) analysis pipeline**

The following describes the various computational steps used in this project for accessing and analyzing the poly(A) tags generated for this project. All steps may be executed on a laptop or desktop computer, and involve the use of commercial software (CLC Genomics Workbench – Qiagen – and Microsoft Excel) as well as freely-available command-line tools run in a Unix framework.

To begin with, it is helpful to review the generic structure of the poly(A) tags generated using Method 2A from Ma et al. [1]. These tags were prepared using reverse transcriptase and primers with the following composition:

5’- (Illumina-compatible sequence) – NNXXXT(18)VN

where NN is a random dinucleotide, XXX is a three-base bar code, and NV a two-base anchor to place the primer at the poly(A)-mRNA junction. The primers used for this project are listed in the table at the end of this document. After sequencing, the first bases read will be the “NN”, followed by the bar code, the oligo-dT tract, and finally the cDNA sequence.

**A. Demultiplex and trim**

The raw data, downloaded in fastq format, are imported into the .clc file format for use with CLC Genomics Workbench. For this, the Illumina import option is used, keeping all of the default settings. Individual sequencing samples are extracted from these files using the Demultiplex Reads option. For this, a two nt linker, followed by the three nt bar code appended to a tract of four T’s, was used. (e.g., the bar code used in the tool will be “XXXTTTT”.) The result of this is a set of files defined by the individual bar codes; note that this process removes the linker and bar codes. These files are then trimmed using the Trim Sequences tool; the Trim Library consists of one sequences – the Illumina PE2 sequence (to remove any Illumina adapter sequences from the 3’-end of the PATs; see the table at the end of the file for this sequence) – that is used with the “Remove Homopolymers” tool that will remove the oligo-dT tract from the 5’ end of each read. What remains are just the cDNA sequences; for each PAT, the 5’-most base corresponds to the 3’ end of the complementary mRNA.

**B. Mapping PATs to the *Sarcocystis neurona* and *Bovine taurus* genomes**

The demultiplexed and trimmed PATs are mapped to the *S. neurona* (SN3DB-35, obtained from Toxo DB – www.toxodb.org) or *B. taurus* (Hereford; ARS-UCD1.2) genomes using the Map Reads to Reference tool in CLC Genomics Workbench. For the mapping, the following parameters were used:

Match score = 1

Mismatch cost = 2

Select Linear gap cost

Length fraction = 0.9

Similarity fraction = 0.9

Global alignment = No

Non-specific match handling = Map randomly

Output mode = Create stand-alone read mappings or Create reads tracks

Create report = Yes

Collect un-mapped reads = No

In addition, for the *S. neurona* mappings, genomic positions corresponding to tracts of 6 or more A’s were masked; this was done to eliminate possible instances of internal priming by reverse transcriptase. For this, a masking track was created (N_6_A_8_) that locates tracts of 8 or more A’s.

These mappings were used to generate variations of the genome browser views presented in the figures in this report. In addition, the results for each bar code (as well as for mappings in which all demultiplexed and trimmed sequences were pooled and used) were exported in bam file format for subsequent processing as described in the following.

**C. Creating a master list of mapped and trimmed PATs**

Unless otherwise indicated, these steps were performed using the Bedtools suite of programs [2]. When completed, this generates a complete list of mapped tags reduced to the genomic coordinates that correspond to the 3’ ends of the individual mRNAs.

1. Convert to bed file:

.bamtobed -i <input bam file> ><output file, .bed format>

2. Trim tags to poly(A) sites – this step utilizes a custom tool that converts the chromosomal coordinates for the mapped reads to one nt tags:

tagtrim <input file, bed format (from step B.1)> <output file, bed format>

(This tool – tagtrim – can be obtained upon request – contact Dr. Hunt at [aghunt00@uky.edu](mailto:aghunt00@uky.edu) . Alternately, it can be downloaded at <https://github.com/ArthurGHunt/tagtrim> .)

3. Sort trimmed tags

.sortbed -i <input file, trimmed tags from step B.2, bed format> ><output file, bed format>

**D. Create a master list of poly(A) clusters (PACs)**

1. Beginning with the output from step C.3, create a PAC list using Bedtools. For this, the distance between adjacent clusters was set to 10 nts:

mergebed -s -d 10 -c 2,6 -o count,distinct -i <input.bed>><output.txt>

2. In Excel, convert the output from step D.1 to bed file format and filter according to total PAT numbers (10).

3. Revise/annotate the PAC list – the idea is to attach gene ID’s and genomic regions (CDS, intron, etc.) to each PAC:

mapbed -S -c 3,9 -o collapse -a <input file in bed format> -b <SN3DB35 regions.gff ><output _PAC24_PAT20_region.txt>

Here, modified gff files (for the *S. neurona* and *B. taurus* genomes) in which all 3’ UTRs have been extended by 500 nts is used. In Excel, rearrange the columns to get to .bed format – I replace the “*” column with gene IDs (from column 9 in the gff file) and the tag numbers with genomic regions (from column 3 in the gff file):

Chr1 5737 5980 <gene_id> three_prime_UTR -

Also, sort the file on columns A and B (smaller to larger). To help keep track of things, it is helpful to include the term “annotated” somewhere in the file name to indicate what has been done. Save in Windows text format, change the suffix to .bed.

**Section II.** **Using DEXSeq to assess alternative polyadenylation**

This section describes the steps used in the Hunt laboratory to adapt the DEXSeq package for the analysis of alternative polyadenylation. Briefly, this pipeline utilizes CLC mappings of poly(A) tags to generate a list of poly(A) site clusters, it generates a dedicated .gff file using these clusters that is appropriate for use with DEXSeq [3], and it generates the .txt files needed by DEXSeq to run the program.

1. Generating the .gtf and .gff flattened files

Using Excel, the PAC list from Step I.D.1 above is converted to the proper .gtf format, yielding a file with this format:

Chr1 AGH exon 361551 361588 . + . gene_id=xxx;transcript_id=xxx

(Please read the DEXSeq documentation for details about the formatting of this file; the format of the last column is very important and cannot be deviated from.)

This file is used to generate the flattened file used in DEXSeq:

python /Users/art/DEXSeq/inst/python_scripts/dexseq_prepare_annotation.py <output_PAC24_PAT10.gtf> <output.DEXSeq.gff>

2. Generating the .txt files

The trimmed and sorted PATSeq reads from Step I.C.3 above are converted to a format suited for use with DEXSeq with the following pipeline:

bedtobam -i *<trimmed and sorted reads in bed format>* -g <organism_name.genome > *<trimmed and sorted reads in bam file format>*

samtools view *<trimmed and sorted reads in bam file format>* > *<trimmed and sorted reads in sam file format>*

awk -F "\t" '{$11=$11"\t\NH:i:1"}1' OFS="\t" *<trimmed and sorted reads in sam file format>* > *<modified reads in sam file format>*

python /Users/art/DEXSeq/inst/python_scripts/dexseq_count.py <output.DEXSeq.gff> *<modified reads in sam file format> <DEXSeq-formatted text file>*

At this point, the data files are ready for DEXSeq. DEXSeq is run in R, using RStudio. Read the DEXSeq documentation for explicit instructions.

**Section III. Analysis of gene expression using CLC Genomics Workbench**

Gene expression was determined using the RNA-Seq Analysis tool in CLC Genomics Workbench. After demultiplexing and trimming (Section I.A above), reads were mapped to the *S. neurona* or *B. taurus* genomes using the “Genome annotated with genes only” option, without spike-in controls. Mapping settings were:

Mismatch cost = 2

Insertion cost = 3

Deletion cost = 3

Length fraction = 0.9

Similarity fraction = 0.9

Global alignment = No

Maximum number of hits for a read = 10

Strand setting = reverse

Library type setting = 3’ sequencing

Expression value = total counts

Other analyses were performed using the Differential Expression for RNA-Seq and PCA for RNA-Seq tools in CLC Genomics Workbench. Results were exported as Excel or PDF files and presented in figures or Supplemental files.

**References**

1. Ma, L., et al., *High throughput characterizations of poly(A) site choice in plants.* Methods, 2014. **67**(1): p. 74-83.

2. Quinlan, A.R., *BEDTools: The Swiss-Army Tool for Genome Feature Analysis.* Curr Protoc Bioinformatics, 2014. **47**: p. 11 12 1-34.

3. Anders, S., A. Reyes, and W. Huber, *Detecting differential usage of exons from RNA-seq data.* Genome Res, 2012. **22**(10): p. 2008-17.
