# Supplementary material for: Transcriptional dynamics in the protozoan parasite Sarcocystis neurona and mammalian host cells after treatment with a specific inhibitor of apicomplexan mRNA polyadenylation
Source: PLoS One. 2021 Oct 28;16(10):e0259109. doi: 10.1371/journal.pone.0259109 (PMC8553156; doi:10.1371/journal.pone.0259109)
Supplement: Attachment — Submitted filename: Plos one Review.docx [file pone.0259109.s010.docx]

This manuscript describes results of *S. neurona* and *N. caninum* treated with AN3661. Similar to *T. gondii* and *P. falciparum*, AN3661-resistant *S. neurona* had mutations in the *cpsf* gene. The study examines the effect of AN3661 on poly(A) sites and transcriptional changes in *S. neurona*. There are a few suggestions to improve the manuscript, below:

Major points:

1. Figure 1: How many times was this experiment done? There are no error bars here.
2. Fig 1A: % inhibition is calculated compared to control wells containing no drug, which I assume would be 0% inhibition. Why does the curve in panel A (S. neurona) not reach 0% inhibition?
3. Fig 1B: There are no data points around the 50% inhibition range. The data points around the 50% inhibition mark is at 5% and then it jumps to ~98% inhibition. This does not allow for accurate IC50 determination. It looks like there are only 5 concentrations examined. This data would be improved if you added more concentrations, especially around the 50% range.
4. For growth inhibition, parasites were only exposed for 2 h before washing out, and 90 nM AN3661 inhibited 100% when examined 4 days later, but measurements were taken daily? This is confusing.
5. It is unclear how AN3661-R *S. neurona* parasites were obtained. They were selected with 90 nM AN36661 – from Fig 1 this inhibits 100%? How long was 90nM AN3661 put on AN3661? Not described in materials and methods section.
6. Growth inhibition studies parasites were exposed to 90nM AN3661 for 2h and 4 days later there was no growth. But for poly(A) site profiling parasites were exposed to 90 nM AN3661 for 24hrs. Parasites are then harvested and examined for poly(A) site profiling. Why this concentration and this time frame? It seems that the parasites would be “in the process of dying” – i.e. if you let it go for a couple more days (without drug) the parasites would die (as you show for growth inhibition).
7. How many biological replicates were performed for poly(A) site profiling?
8. Fig 3 legend: how long were *S. neurona* and *B. taurus* treated for with 90 nM AN3661? Why was this drug concentration chosen – this inhibits Sn at 100% (Fig 1)
9. Fig 5: what do the error bars represent? Not described in the legend
10. Fig 6: why is LDH positive control twice as much as “max LDH release from lysed cells”? Why are the bar graphs of different width?
11. Fig 8: what do the error bars represent? Not described in the legend
12. Whole genome seqencing was not performed on AN3661-resistant parasites, but rather the researchers amplified the *cpsf* gene and then sequenced the gene. Therefore would be good to mention in discussion that there could be other genetic mutations underlying AN3661-R.

Minor points:

1. Lines 34-35: this sentence is confusing. If vaccines are ineffective against coccidian parasite how is this an option to reduce coccidiosis?
2. Line 40: EPM not defined
3. Line 103: RPM (typo)
4. The figure legends are peppered in the body of the manuscript.
5. After the first mention of *Sarcocystis neurona*, future mentions should be *S. neurona*
6. Line 162: should be bovine turbinate (BT) not bovine (BT) turbinate
7. Line 192: FDR is 0.05 while Line 271 refers to false discovery rate adjusted p-value (i.e. q-value) less than 0.05. BT analysis refers to analysis done on Sn. Was the same analyses performed? Please clarify.
8. Would be good to define “Adjusted p-value” vs :p-value”
