# Supplementary material for: Transcriptional dynamics in the protozoan parasite Sarcocystis neurona and mammalian host cells after treatment with a specific inhibitor of apicomplexan mRNA polyadenylation
Source: PLoS One. 2021 Oct 28;16(10):e0259109. doi: 10.1371/journal.pone.0259109 (PMC8553156; doi:10.1371/journal.pone.0259109)
Supplement: Attachment — Submitted filename: Hunt et al. Response to Review.docx [file pone.0259109.s011.docx]

To the Editor,

The following summarizes changes and other discussion regarding the review of manuscript PONE-D-21-14533, “Transcriptional dynamics in the protozoan parasite Sarcocystis neurona and mammalian host cells after treatment with a specific inhibitor of apicomplexan mRNA polyadenylation”, that we have submitted for publication in PLOS ONE. This document is organized using the reviewing outline and format. In so doing, our changes, responses, and other discussion are set apart as indented text.

Reviewers' comments:

Reviewer's Responses to Questions

**Comments to the Author**

1. Is the manuscript technically sound, and do the data support the conclusions?

The manuscript must describe a technically sound piece of scientific research with data that supports the conclusions. Experiments must have been conducted rigorously, with appropriate controls, replication, and sample sizes. The conclusions must be drawn appropriately based on the data presented.

Reviewer #1: Yes

Reviewer #2: Partly

As indicated in the detailed response, we have made changes to satisfy the concerns regarding statistical analyses and presentation of some data items.

Reviewer #3: Yes

2. Has the statistical analysis been performed appropriately and rigorously?

Reviewer #1: No

As indicated in the detailed response, we have made changes to satisfy the concerns regarding statistical analyses and presentation of some data items.

Reviewer #2: No

As indicated in the detailed response, we have made changes to satisfy the concerns regarding statistical analyses and presentation of some data items.

Reviewer #3: Yes

3. Have the authors made all data underlying the findings in their manuscript fully available?

The [PLOS Data policy](https://nam04.safelinks.protection.outlook.com/?url=http%3A%2F%2Fwww.plosone.org%2Fstatic%2Fpolicies.action%23sharing&data=04%7C01%7Caghunt00%40uky.edu%7C79b844f8a49e49599f4508d937426b8c%7C2b30530b69b64457b818481cb53d42ae%7C0%7C0%7C637601581873985711%7CUnknown%7CTWFpbGZsb3d8eyJWIjoiMC4wLjAwMDAiLCJQIjoiV2luMzIiLCJBTiI6Ik1haWwiLCJXVCI6Mn0%3D%7C1000&sdata=Uh2S0hMW4mulHDdWoL%2BakjI%2BG5If1JL7GDVzvsi%2Bk9k%3D&reserved=0) requires authors to make all data underlying the findings described in their manuscript fully available without restriction, with rare exception (please refer to the Data Availability Statement in the manuscript PDF file). The data should be provided as part of the manuscript or its supporting information, or deposited to a public repository. For example, in addition to summary statistics, the data points behind means, medians and variance measures should be available. If there are restrictions on publicly sharing data—e.g. participant privacy or use of data from a third party—those must be specified.

Reviewer #1: Yes

Reviewer #2: Yes

Reviewer #3: Yes

4. Is the manuscript presented in an intelligible fashion and written in standard English?

PLOS ONE does not copyedit accepted manuscripts, so the language in submitted articles must be clear, correct, and unambiguous. Any typographical or grammatical errors should be corrected at revision, so please note any specific errors here.

Reviewer #1: Yes

Reviewer #2: Yes

Reviewer #3: Yes

5. Review Comments to the Author

Please use the space provided to explain your answers to the questions above. You may also include additional comments for the author, including concerns about dual publication, research ethics, or publication ethics. (Please upload your review as an attachment if it exceeds 20,000 characters)

Reviewer #1: The study by Hunt et al. shows the effects of treating Sarcocystis neurona and Neospora caninum with a known inhibitor of the apicomplexan polyadenylation complex. The study is technically sound and underlying sequencing data have been deposited to a public repository. Results of bioinformatics analyses are mostly included in well-documented Supplementary Tables. Before publication, the authors should appropriately analyse the IC50 data and include replicate number. The differential gene expression analysis (as presented in Figure 3B) should also be explained in the Materials and Methods - it was not clear from the methods in Supplementary File S2 how this was performed after read mapping.

We have added the requested information to the Supplementary File S2, as an additional section (Section III). This addition should satisfy the request. Additional text was not added to the Materials and Methods, since this would be redundant. (File S2 has been further edited to fix some other issues and hopefully make it easier to follow.)

The assessment of growth inhibition, including replicate numbers, has been clarified in the Materials and Methods, Results, and the Figure legend, and IC50 values for S. neurona have now been more appropriately determined by regression analysis. Since it was a minor component of the study and the data were not strong, the Neospora growth assay and IC50 determination have been removed from the manuscript.

I would also suggest moving some of Fig S1 to the main Figures, to complement visualization of the data represented in Figure 5.

After trying to add some browser tracks to Fig. 5, we find that the resulting revised figure is very difficult to read, because of the different formats and styles for the charts and browser tracks. Thus, we have decided to leave Fig. 5 and Fig. S1 unchanged.

Major comments

Fig1A and 1B: The IC50 curves have inconsistent curved lines between data points. The authors should fit an IC50 regression and calculate the IC50 from that.

Our presentation of the growth assay results (Fig. 1) has been revised, and the IC50 for S. neurona has been calculated by regression analysis. We have decided that the N. caninum growth assays were not pursued adequately. Since these data were a minor component of the study, we have elected to remove the old Figure 1B. The new Figure 1B shows results of a *S. neurona* growth assay using a more narrow range of AN3661 concentrations to better estimate the IC50.

Minor comments

Lines 101 and 103: Should consistently use g instead of RPM (typo RMP on line 103)

RPM has been replaced with x g.

Line 108: Specify that the data are available at the NCBI Sequencing Read Archive under the Bioproject accession PRJNA713353

This change has been made as requested (lines 119-120).

Line 116: The Supplemental File 2 docx has the heading “Supplemental File 3”

This error has been corrected.

Line 133: Were biological replicates performed for the growth assays? This should be specified (eg n = 1, or n = 3) in the Figure 1 legend. If n >1 error bars should be added or individual datapoints plotted.

The number of assays and replicates has been clarified in the figure legend (lines 154-155) and the Materials and Methods (lines 74-79), and the figure has been revised as recommended.

Line 143: convention to denote mutation as (for example) Y668N, but using residue numbering for the S. neurona CPSF73.

Figure 2 has been revised to account for this, and to add residue numbers appropriate for each sequence that was analyzed.

Line 150: There are some residues in black that are not identical between all 5 sequences, eg residue 519. Change the Figure 2 legend to reflect this.

The legend to Fig. 2 has been revised to better explain the residue shading that was used to depict the alignment.

Figure 7: The tick labels are too blurry to read

The font size for the tick legends have been increased to allow for easier reading.

Line 518: Italicise species names

This correction has been made.

Line 539: remove COI disclosure

This correction has been made.

S2 File: To enable reproducibility, add the code for “tagtrim” to the S2 File or a public repository such as GitHub.

The “tagtrim” program has been deposited on Github and links provided.

Reviewer #2: This manuscript describes results of S. neurona and N. caninum treated with AN3661. Similar to T. gondii and P. falciparum, AN3661-resistant S. neurona had mutations in the cpsf gene. The study examines the effect of AN3661 on poly(A) sites and transcriptional changes in S. neurona. There are a few suggestions to improve the manuscript, below:

Major points:
1. Figure 1: How many times was this experiment done? There are no error bars here.

The number of assays and replicates has been clarified in the text (lines 140-150), figure legend (lines 154-155) and the Materials and Methods (lines 74-79), and the figure has been revised as recommended.

2. Fig 1A: % inhibition is calculated compared to control wells containing no drug, which I assume would be 0% inhibition. Why does the curve in panel A (S. neurona) not reach 0% inhibition?

Although these data have been re-plotted and the figure revised, the principle is the same. The reviewer is correct that the % inhibition (now % growth) in the treatment wells is relative to the no-drug control wells. Consequently, the control wells are not plotted in the graph; only the treatment data are plotted. Since some inhibition was observed even at the lowest concentration, the curve does not reach 0% inhibition (now 100% growth).

3. Fig 1B: There are no data points around the 50% inhibition range. The data points around the 50% inhibition mark is at 5% and then it jumps to ~98% inhibition. This does not allow for accurate IC50 determination. It looks like there are only 5 concentrations examined. This data would be improved if you added more concentrations, especially around the 50% range.

The reviewer is correct that the growth assay data for Neospora (Fig.1B) was not sufficient. Since Sarcocystis is the primary focus of the study, we have removed the Neospora results. A new Figure 1B is now presented, which shows the growth curve for S. neurona over a more narrow range of drug concentrations.

4. For growth inhibition, parasites were only exposed for 2 h before washing out, and 90 nM AN3661 inhibited 100% when examined 4 days later, but measurements were taken daily? This is confusing.

We apologize for the lack of clarity. The growth assays were conducted with the AN3661 drug present for the duration of the 4-day growth period, not just during the initial 2-hour incubation when parasites were allowed to invade the host cells. This has been clarified in the Materials and Methods (lines 74-87).

Although the plates were monitored daily, only the data from day 4 are presented in Figure 1 and used for the IC50 calculations. This has been clarified in the Materials and Methods (lines 74-87).

5. It is unclear how AN3661-R S. neurona parasites were obtained. They were selected with 90 nM AN36661 – from Fig 1 this inhibits 100%? How long was 90nM AN3661 put on AN3661? Not described in materials and methods section.

The mutagenized population was grown in the presence of AN3661 for approximately 5 weeks, followed by continued selection during the single-cell cloning. This has been clarified in the Materials and Methods (lines 90-96). As mentioned by the reviewer, 90nM was chosen because our growth assays (e.g., Fig. 1) showed that this fully inhibited parasite growth (now mentioned in the Results section, lines 164-167).

6. Growth inhibition studies parasites were exposed to 90nM AN3661 for 2h and 4 days later there was no growth. But for poly(A) site profiling parasites were exposed to 90 nM AN3661 for 24hrs. Parasites are then harvested and examined for poly(A) site profiling. Why this concentration and this time frame? It seems that the parasites would be “in the process of dying” – i.e. if you let it go for a couple more days (without drug) the parasites would die (as you show for growth inhibition).

As clarified above for item #4, the AN3661 drug was present for the entire 4-day growth assay, not just during the initial 2-hour incubation. It is believed that AN3661 is not a parasiticidal drug, but rather just inhibits parasite growth via disruption of normal RNA polyadenylation (the intent of the transcriptome experiment). As we mention above for item #5, 90 nM was chosen because it fully inhibited parasite growth and we wanted to ensure large differences in poly(A) profiling between the treatment sample and the non-treated control sample.

7. How many biological replicates were performed for poly(A) site profiling?

The Materials and Methods (lines 102-109) have been edited to make it clear that three biological replicates were prepared for each condition in the poly(A) site profiling.

8. Fig 3 legend: how long were S. neurona and B. taurus treated for with 90 nM AN3661? Why was this drug concentration chosen – this inhibits Sn at 100% (Fig 1)

As indicated in the Materials and Methods, the AN3661 treatments were done for 24 hr. 90 nM was chosen for the AN3661 concentration for the poly(A) site profiling so as to assure that the two populations (treated and control) were distinct. Use of a concentration nearer the LD50 could have resulted in a mixed population of cells exhibiting different extents of inhibition and transcriptome alterations.

9. Fig 5: what do the error bars represent? Not described in the legend

The error bars represent standard deviations calculated from values in each of the replicates. The figure legend has been edited to make this clear

10. Fig 6: why is LDH positive control twice as much as “max LDH release from lysed cells”? Why are the bar graphs of different width?

The LDH positive control is an enzyme control provided by the manufacturer to ensure that the assay has been conducted properly. The maximum release sample represents the same number of BT cells as those that have been treated and, therefore, shows the level of LDH release that would be seen if a drug was highly cytotoxic. We neglected to include a description of the LDH release assay in the Materials and Methods section. This has been added (lines 128-135). We have also revised the results and Fig. 6 legend to better explain the results of this experiment.

11. Fig 8: what do the error bars represent? Not described in the legend

The error bars represent standard deviations calculated from values in each of the replicates. The figure legend has been edited to make this clear.

12. Whole genome sequencing was not performed on AN3661-resistant parasites, but rather the researchers amplified the cpsf gene and then sequenced the gene. Therefore would be good to mention in discussion that there could be other genetic mutations underlying AN3661-R.

This point has been raised in the revision (lines 172-175).

Minor points:
1. Lines 34-35: this sentence is confusing. If vaccines are ineffective against coccidian parasite how is this an option to reduce coccidiosis?

This sentence has been revised to specify that vaccination is effective against poultry coccidiosis caused by Eimeria, but has not been effective against other species of coccidia.

2. Line 40: EPM not defined

This has been changed to Equine Protozoal Myeloencephalitis.

3. Line 103: RPM (typo)

This error has been corrected.

4. The figure legends are peppered in the body of the manuscript.

This is the format required for submissions to PLoS ONE.

5. After the first mention of Sarcocystis neurona, future mentions should be S. neurona

This correction has been made.

6. Line 162: should be bovine turbinate (BT) not bovine (BT) turbinate

This correction has been made.

7. Line 192: FDR is 0.05 while Line 271 refers to false discovery rate adjusted p-value (i.e. q-value) less than 0.05. BT analysis refers to analysis done on Sn. Was the same analyses performed? Please clarify.

This statement has been corrected, as suggested. The parameter used in these analyses was indeed the q-value.

8. Would be good to define “Adjusted p-value” vs :p-value”

Assuming that this refers to the description of the poly(A) site analyses (lines 237-239 and 303-306), the terms “FDR-adjusted p-value” and “raw p-value” have been inserted where needed.

Reviewer #3: The manuscript is exceedingly well-written and well-organized with sufficient detail to support scientific claims.

I have only a few comments/questions/suggested edits:

1. The information provided in the introduction could be better supported with citations.

An additional reference that succinctly reviews the field had been added.

2. Line 52 - affective should be effective

This correction has been made.

3. Sequencing of the SnCPSF73 gene of the AN3661-resistant clones was performed. Did the authors consider sequencing the full genome of the clones to ensure no other mutant genes were contributing to the phenotype?

We have not done whole-genome sequencing, largely because of cost considerations. Were this the first study of AN3661-resistant apicomplexans, we would concur that whole-genome sequencing and a more exhaustive ruling out of other changes would be called for. However, it is well-established that the changes we report are responsible for AN3661 resistance in other apicomplexans; the consistency we see in multiple mutant isolates suffices to confirm the hypothesis, in our opinion.

4. Line 294 - Typo "BY" host cells, should be "BT"

This error has been corrected.

5. Line 351 - typo "t="

This error has been corrected.

6. Figure 2 - What does the blue star indicate?

These were inadvertent additions to the figure and do not belong. They are carryovers from a different version of the figure, intended to highlight features not discussed in this study.

7. Figure 3B. x/y axis text not legible, poor resolution.

The figure has been modified to improve the legibility and resolution.

8. figure 8. (Line 437) Should this be BT cells instead of BY?

This error has been corrected.

9. Figure 8. - Does the "I" in BTI stand for anything? Inhibitor? Because there aren't any parasites in this population of cells consider changing for clarity (e.g., BT-A90).

We believe the term “BTI” is adequately defined in the legend, so this has not been changed.

6. PLOS authors have the option to publish the peer review history of their article ([what does this mean?](https://nam04.safelinks.protection.outlook.com/?url=https%3A%2F%2Fjournals.plos.org%2Fplosone%2Fs%2Feditorial-and-peer-review-process%23loc-peer-review-history&data=04%7C01%7Caghunt00%40uky.edu%7C79b844f8a49e49599f4508d937426b8c%7C2b30530b69b64457b818481cb53d42ae%7C0%7C0%7C637601581873995664%7CUnknown%7CTWFpbGZsb3d8eyJWIjoiMC4wLjAwMDAiLCJQIjoiV2luMzIiLCJBTiI6Ik1haWwiLCJXVCI6Mn0%3D%7C1000&sdata=spNAP4lkifCqVgcVKLNFEmPy7zXMMVqf0%2BWnFP21Myc%3D&reserved=0)). If published, this will include your full peer review and any attached files.


If you choose “no”, your identity will remain anonymous but your review may still be made public.


**Do you want your identity to be public for this peer review?** For information about this choice, including consent withdrawal, please see our [Privacy Policy](https://nam04.safelinks.protection.outlook.com/?url=https%3A%2F%2Fwww.plos.org%2Fprivacy-policy&data=04%7C01%7Caghunt00%40uky.edu%7C79b844f8a49e49599f4508d937426b8c%7C2b30530b69b64457b818481cb53d42ae%7C0%7C0%7C637601581873995664%7CUnknown%7CTWFpbGZsb3d8eyJWIjoiMC4wLjAwMDAiLCJQIjoiV2luMzIiLCJBTiI6Ik1haWwiLCJXVCI6Mn0%3D%7C1000&sdata=7sa8XUeJ94aM2I%2BzkY2MjnPEluhE3QZBd4xTfVAtCUM%3D&reserved=0).

Reviewer #1: No

Reviewer #2: **Yes:** Caroline Ng

Reviewer #3: No

[NOTE: If reviewer comments were submitted as an attachment file, they will be attached to this email and accessible via the submission site. Please log into your account, locate the manuscript record, and check for the action link "View Attachments". If this link does not appear, there are no attachment files.]

While revising your submission, please upload your figure files to the Preflight Analysis and Conversion Engine (PACE) digital diagnostic tool, [https://pacev2.apexcovantage.com/](https://nam04.safelinks.protection.outlook.com/?url=https%3A%2F%2Fpacev2.apexcovantage.com%2F&data=04%7C01%7Caghunt00%40uky.edu%7C79b844f8a49e49599f4508d937426b8c%7C2b30530b69b64457b818481cb53d42ae%7C0%7C0%7C637601581873995664%7CUnknown%7CTWFpbGZsb3d8eyJWIjoiMC4wLjAwMDAiLCJQIjoiV2luMzIiLCJBTiI6Ik1haWwiLCJXVCI6Mn0%3D%7C1000&sdata=cXrtuHLAwIOnFyWrvFspuZpWhqV%2Bquot3zwPs9719Z8%3D&reserved=0). PACE helps ensure that figures meet PLOS requirements. To use PACE, you must first register as a user. Registration is free. Then, login and navigate to the UPLOAD tab, where you will find detailed instructions on how to use the tool. If you encounter any issues or have any questions when using PACE, please email PLOS at [figures@plos.org](mailto:figures@plos.org). Please note that Supporting Information files do not need this step.
