# Supplementary material for: Transcriptional dynamics in the protozoan parasite Sarcocystis neurona and mammalian host cells after treatment with a specific inhibitor of apicomplexan mRNA polyadenylation
Source: PLoS One. 2021 Oct 28;16(10):e0259109. doi: 10.1371/journal.pone.0259109 (PMC8553156; doi:10.1371/journal.pone.0259109)
Supplement: Attachment — Submitted filename: Review.docx [file pone.0259109.s012.docx]

The revised manuscript is much improved, especially in regards to statistical analyses, graphical representations, and clarification of methods. Here are some suggestions to further improve the manuscript, detailed below:

Major Comments:

1. Define abbreviations at first use. Some examples (please go through to identify others):
   a. CPSF73 in the abstract Line 4
   b. BT cells Line 70 Methods
   c. FDR Line 240
   d. YSH1 Line 381
   e. IPA1 Line 383
2. Reference 1 – can’t find this in PubMed. Would be good to include doi for all articles not in PubMed.
3. Statements in the introduction are largely unsupported by the necessary references. Please include a reference for each statement made, e.g. for statements in lines 24-32, lines 38-48, 50-51
4. Methods: Please include more details on the LDH assay – eg what wavelength was this assay read at? What is the composition of the lysis buffer?
5. How many total drug-resistant single-cell clones were obtained? (in reference to lines 167-168)
6. Fig. 4: what does DEGs stand for? What does the blue circle and the yellow circle indicate? Unclear from the figure and legend.
7. “The results (Fig. 4) show that between 24 and 27% of genes 247 whose poly(A) site profiles change also showed significant changes in overall transcript levels.” – I don’t see these percentages in the figure.
8. Fig. 6: What is the measurement that was taken? As written it looks like a measurement was taken at Abs 490 through Abs 680 nm. A quick look into the manufacturer’s protocol suggests that this is not the case. Please correct the figure itself to more accurately reflect what was done, and also describe this in the figure legend.
9. Statement on Lines 418-420 incorrect: studies were performed with various initial populations of *P. falciparum*; the least amount of parasites was 2 x 10^7^

Minor Comments:

1. Line 70: Space between cloneswere
2. Line 81: 37^o^C
3. Be consistent: hours denoted as “h” in line 80 but “hrs” on lines 103,104 and elsewhere
4. Fig 1 legend: Line 158: nM; line 160: IC_50_, and needs units after 14.99.
5. Fig 2: “Hs” is not aligned with the other names. Suggest writing Tg, Sn, Pf, At, Hs for consistency. Information about strains that this sequence comes from should be addressed in the legend. – i.e. please explain TGME49, SN3, Pf3D7
6. Line 193 – (BT) after “bovine turbinate” and not “bovine”
7. Line 340: benzoxaborole (typo)
8. Line 427: no studies on *N. caninum* anymore in this report
9. Fig 3 legend: *S. neurona* and *B. taurus* need to be italicized
