# Supplementary material for: Transcriptional dynamics in the protozoan parasite Sarcocystis neurona and mammalian host cells after treatment with a specific inhibitor of apicomplexan mRNA polyadenylation
Source: PLoS One. 2021 Oct 28;16(10):e0259109. doi: 10.1371/journal.pone.0259109 (PMC8553156; doi:10.1371/journal.pone.0259109)
Supplement: Attachment — Submitted filename: PONE Review and Response 092921.docx [file pone.0259109.s013.docx]

To the Editor,

The following summarizes changes and other discussion regarding the second review of manuscript PONE-D-21-14533, “Transcriptional dynamics in the protozoan parasite Sarcocystis neurona and mammalian host cells after treatment with a specific inhibitor of apicomplexan mRNA polyadenylation”, that we have submitted for publication in PLOS ONE. This document is organized using the reviewing outline and format. In so doing, our changes, responses, and other discussion are set apart as indented text.

Major Comments:

1. Define abbreviations at first use. Some examples (please go through to identify others):
   a. CPSF73 in the abstract Line 4
   b. BT cells Line 70 Methods
   c. FDR Line 240
   d. YSH1 Line 381 – no need to change, since this is a gene name
   e. IPA1 Line 383 - no need to change, since this is a gene name

The changes in lines 4, 70, and 240 of the first revision have been made as suggested. USH1 and IPA1 are formal gene designations and not abbreviations. Accordingly, these have not been changed or further defined.

2. Reference 1 – can’t find this in PubMed. Would be good to include doi for all articles not in PubMed.

The doi for this book has been added. We believe the other references are in Pubmed.

3. Statements in the introduction are largely unsupported by the necessary references. Please include a reference for each statement made, e.g. for statements in lines 24-32, lines 38-48, 50-51

We appreciate this remark. However, these statements are all general parasitology textbook information (as could be found in the first reference). We would argue that references to the literature, or other textbooks, are appropriate for this paragraph. Accordingly, we have not added any.

4. Methods: Please include more details on the LDH assay – eg what wavelength was this assay read at? What is the composition of the lysis buffer?

This section of the Methods has been updated as requested. We would note that the lysis buffer was provided with the commercial assay. The composition of the buffer is not provided in the documentation by the manufacturer.

5. How many total drug-resistant single-cell clones were obtained? (in reference to lines 167-168)

This statement has been updated as requested.

6. Fig. 4: what does DEGs stand for? What does the blue circle and the yellow circle indicate? Unclear from the figure and legend.

The legend to Figure 4 has been edited so as to clarify these questions. In particular, in the legend we now note that the yellow circles (identifiable as “DEG” in the figure) corresponds to differentially-expressed genes.

7. “The results (Fig. 4) show that between 24 and 27% of genes 247 whose poly(A) site profiles change also showed significant changes in overall transcript levels.”

We have not altered the figure, out of the belief that to add percentages to the figure in addition to or instead of the total gene numbers would be confusing, and would not allow readers to appreciate the scope of the changes we describe. It is relatively simple for a reader to divide the numbers in the overlap by the numbers of total genes in each class, to come to the percentages we mention.

8. Fig. 6: What is the measurement that was taken? As written it looks like a measurement was taken at Abs 490 through Abs 680 nm. A quick look into the manufacturer’s protocol suggests that this is not the case. Please correct the figure itself to more accurately reflect what was done, and also describe this in the figure legend.

We have corrected the Y axis label and defined the terms in the revised figure legend..

9. Statement on Lines 418-420 incorrect: studies were performed with various initial populations of *P. falciparum*; the least amount of parasites was 2 x 10^7^ .

From Ref 7: “We quantified the ease of *in vitro* selection of resistance to AN3661 by subjecting 10^6^–10^8^ Dd2 strain parasites to 60 nM (2 × IC_90_) AN3661. Regrowth was seen in two of three cultures with initial inocula of 10^6^ parasites at days 45 and 56, one of three cultures with initial inocula of 10^7^ parasites at day 23, and three of three cultures with initial inocula of 10^8^ parasites, all on day 19 ([Supplementary Table 4](https://www.ncbi.nlm.nih.gov/pmc/articles/PMC5343452/#S1)). These rates of resistance selection were similar to those observed for atovaquone.”

Minor Comments:

1. Line 70: Space between cloneswere

This correction has been made.

2. Line 81: 37^o^C

This correction has been made.

3. Be consistent: hours denoted as “h” in line 80 but “hrs” on lines 103,104 and elsewhere

We now use hrs throughout..

4. Fig 1 legend: Line 158: nM; line 160: IC_50_, and needs units after 14.99.

These corrections have been made.

5. Fig 2: “Hs” is not aligned with the other names. Suggest writing Tg, Sn, Pf, At, Hs for consistency. Information about strains that this sequence comes from should be addressed in the legend. – i.e. please explain TGME49, SN3, Pf3D7

The figure has been slightly modified, and the legend edited, to address this issue. We would note that the gene identifiers for the proteins has been added to the Methods.

6. Line 193 – (BT) after “bovine turbinate” and not “bovine”

This correction has been made.

7. Line 340: benzoxaborole (typo).

This correction has been made.

8. Line 427: no studies on *N. caninum* anymore in this report

This correction has been made.

9. Fig 3 legend: *S. neurona* and *B. taurus* need to be italicized

This correction has been made.
